# Enhancing gain and isolation of a quad-element MIMO antenna array design for 5G sub-6 GHz applications assisted with characteristic mode analysis

**DOI:** 10.1038/s41598-024-61789-7

**Published:** 2024-05-15

**Authors:** Rabia Khan, Waleed Tariq Sethi, Waqar Ahmad Malik, Latif Jan, Muhammad M. Tahseen, Ali M. Almuhlafi, Mohamed Himdi

**Affiliations:** 1 Faculty of Electrical Engineering, Ghulam Ishaq Khan Institute of Engineering Sciences and Technology, Swabi, 23640 Pakistan; 2 Department of Avionics Engineering, College Aeronautical Engineering (CAE), National University of Sciences and Technology (NUST), Risalpur, KPK Pakistan; 3 Department of Computer Science, Iqra National University, Peshawar, 25100 Pakistan; 4 School of Electrical Engineering and Computer Science (SEECS), National University of Sciences and Technology (NUST), Islamabad, 44000 Pakistan; 5 Electrical Engineering Department, King Saud University, 11421 Riyadh, Saudi Arabia; 6 Institut d’Electronique et des Technologies du numeRique (IETR), University of Rennes, 35042 Rennes, France

**Keywords:** 5G, Characteristic mode analysis (CMA), MIMO antenna array, Quad-element, Sub-6 GHz, Engineering, Electrical and electronic engineering

## Abstract

This paper presents a novel quad-element array with multiple inputs and multiple outputs (MIMO) designed for 5th generation sub-6 GHz applications. The MIMO system achieves a wide impedance bandwidth, high gain, and high isolation among its components, representing significant advancements in sub-6 GHz antenna applications. The single element, an elliptical resonator with a circular slot, is fed by a 50 Ω microstrip feedline, achieves a broad characteristic bandwidth from 3.7 to 5.7 GHz with a resonant frequency of 4.33 GHz and a gain of 1.81 dBi. Characteristic Mode Analysis (CMA) was employed to elucidate the evolution phases of this design. The quad-element MIMO antenna array maintains a compact size and broadband characteristics by arranging mirrored elements on the same ground plane. Implemented on a cost-effective FR-4 substrate measuring 44 × 44 × 1.6 mm^3^, the recommended MIMO antenna array, enhanced with a partial ground plane and due to the introduction of a vertical strip, a high isolation of − 38.53 dB is achieved between MIMO components along with a realized gain of 3.01 dBi and a radiation efficiency of 71% in the 5G sub-6 GHz band. Noteworthy properties include high isolation, diversity gain (DG), and envelope correlation coefficient (ECC), verifying the appropriateness of the suggested MIMO scheme for 5G transmission and reception in sub-6 GHz applications.

## Introduction

**Figure 1:**
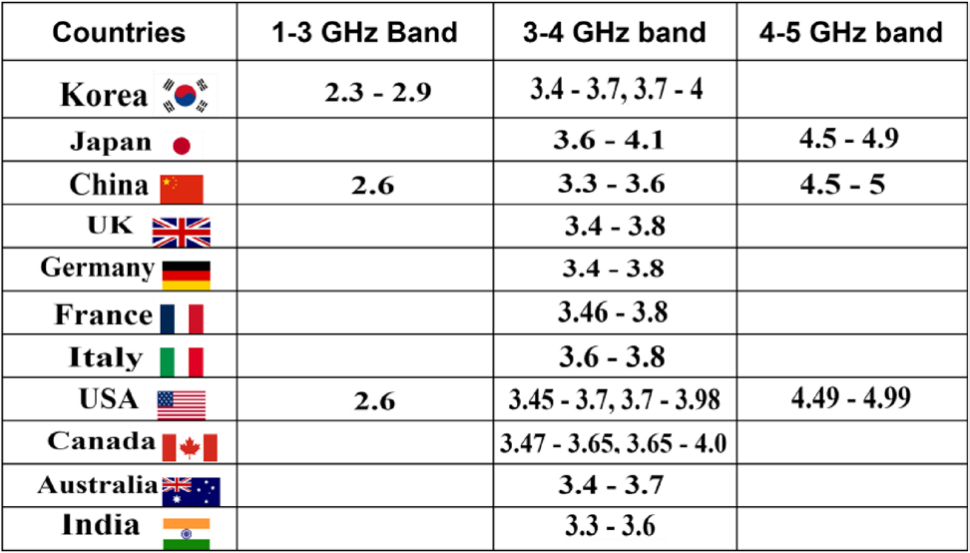
Global spectrum allotment for 5G sub-6 GHz applications^1^.

With the tremendous leap in wireless communication, known as 5G, billions of connected devices now have access to faster and more secure networks, providing low-latency customer experiences and enabling cutting-edge services that are revolutionizing various aspects of our lives, society, and industries, including health informatics, intelligent manufacturing, digital cities, home automation, cyberspace, and the Internet of Things (IoT). One specific band within the 5G spectrum is the sub-6 GHz range, which has garnered significant attention from scientists and experts because of its capability to facilitate high-speed communication over long distances with a high resolution. To achieve the intended performance in the sub-6 GHz range, antennas are essential. The Federal Communication Commission (FCC) divides the available 5G spectrum into four bands. Figure 1 illustrates the distribution of the 5G sub-6 GHz range across global wireless networks, indicating that the sub-6 GHz range for 5G transmissions continues to be considered among all countries^1^.

The MIMO system plays a crucial role in wireless communications; as it makes it possible to transmit data using several antennas at once, resulting in improved data transfer speed, spectral efficiency, channel capacity, reliability, gain, and bandwidth^2,3^. Due to their ability to transmit and receive data over multiple channels with low energy consumption, MIMO antennas are a viable choice for sub-6 GHz applications. One of the primary concerns for researchers is the inter-element coupling effect between MIMO elements, which arises from the proximity of multiple radiating elements within a confined space. In recent years, researchers have reported various techniques to address inter-element coupling, including the use of decoupling elements^4^, shielding walls^5^, electromagnetic band gap (EBG) structures^6^, and meta-surfaces^7^. Decoupling elements are employed to prevent surface currents from flowing between energized ports. However, the inclusion of decoupling elements adds complexity to the design and profile of the MIMO antennas.

In^8^, a MIMO antenna based on eight-elements is introduced having measurements of 68 × 68 × 21.6 mm^3^, resonating at 5.8 GHz with a wide impedance bandwidth of 25.68 GHz, isolation of greater than 18 dB and an Envelope Correlation Coefficient (ECC) of below 0.025. However, the issue of inter-element coupling is addressed through polarization diversity. The radiating components were positioned in both the vertical and horizontal planes to reduce their mutual entanglement, eliminating the need for additional decoupling structures. However, the proposed design is more intricate, non-planar, and physically larger. In^9^, a four-port MIMO design was suggested for services in the 5G New Radio Access Technology (RAT) Wireless Regional Area Network functioning inside the sub-6 GHz range of 3.2–5.75 GHz having measurements of 40 × 40 × 1.6 mm^3^. The resonating frequency is 5.4 GHz with a gain of 9 dBi, radiation efficiency of 90% and ECC is less than 0.05. The antenna components were separated by 0.3*λ* and orthogonally oriented to each other. Various decoupling structures, such as the electromagnetic band gap (EBG), defected ground structure (DGS), capacitive elements (CE), and neutralization lines (NL), are employed amid the components to enhance isolation which is – 45 dB. However, the use of these decoupling structures adds complexity to design. In^10^, a shared design is proposed, featuring a four-port radiator with multiple cuts and a slightly slanted ground for wireless applications at 5.7 GHz. The dimensions of this design are 75 × 75 × 1.6 mm^3^. It offers an isolation of over 13 dB, ECC below 0.04, a gain of 4 dBi, and an impedance bandwidth of 4.4–6.4 GHz. However, the antenna design is large and has a complex structure. In^11^, an orthogonal circular polarization (CP) MIMO antenna is recommended for 5G operations in the sub-6 GHz frequency range. The constituents of MIMO antenna are four radiating components with dimensions of 123 × 123 × 1.6 mm^3^ and a central frequency of 3.7 GHz. Its impedance bandwidth ranges from 3.4 to 3.8 GHz at – 10 dB, with a gain of 5 dBic, an ECC of 0.004, and an isolation of – 20 dB. However, it has a large structure with a relatively narrow impedance bandwidth.

Furthermore, in^12^, a wideband MIMO antenna based on two elements, with a line patch between them, was proposed. It had dimensions of 66 × 66 × 1.6 mm^3^*.* The operating frequency is 2.08 GHz, with an impedance bandwidth of 1.84–2.57 GHz, a gain of 3 dB, and an ECC below 0.01. The two ports exhibit − 24 dB isolation and a nearly 10 dB diversity gain. However, this design has limitations owing to its small bandwidth and bulkiness. In^13^, a miniature planar monopole MIMO antenna operating at a frequency of 3.5 GHz is recommended. The antenna has dimensions of 47.5 × 40 × 1.6 mm^3^. The antenna components’ interaction with each other in the MIMO design is reduced using two broadband metamaterial split-ring resonators. The suggested MIMO antenna configuration provides a bandwidth ranging from to 3.35–3.78 GHz, a gain of 3 dB, over 15 dB of isolation, an ECC below 0.05, and a radiation efficiency of 82%. However, this design has limited bandwidth, and the presence of a decoupling element between the elements adds complexity to the design. In^14^, a 2 × 2 MIMO antenna based on four-ring slot resonators with concentric complementary split-ring resonators (CSRR) is proposed within the sub-6 GHz range. The antenna ports’ coupling was lessened by using three linearly arranged CSRR components. The suggested MIMO antenna’s measurements are 75 mm × 150 × 1.6 mm^3^, with an operating frequency of 3.6 GHz and gain of 3.5 dBi. The design exhibited an isolation of 17 dB, an ECC of 0.03 in the working frequency band of 3.4–3.8 GHz, and a mean effective gain (MEG) of – 3 dB. However, the impedance bandwidth of the antenna was only 0.4 GHz, which is relatively small, and its radiation efficiency was 81% at 3.6 GHz. The MIMO antenna design is intricate owing to the inclusion of a decoupling structure to reduce the inter-element coupling.

In^15^, a dual-port, two-element MIMO antenna design with dimensions of 50 × 30 × 1.6 mm^3^. The design utilizes Transparent Conductive Oxide (AgHT-8) material for the patches and ground, while plexiglass serves as the substrate. Two similar circular radiating components were activated using a microstrip feedline. Two cases are considered in this study. In the first case, the elements were positioned in an inline arrangement, with separate ground profiles. The antenna operated at a frequency of 4.81 GHz with an impedance bandwidth of only 6.65% (4.65–4.97 GHz) and a gain of 1.02 dB. In the first case, the MIMO antenna has an efficiency of 48–53%, a reflection coefficient of − 17.18 dB, and an isolation of over 17 dB. This high isolation is due to the distinct ground profiles of each antenna element. The ECC value was below 0.05, and the diversity gain ranges from 9.88 to 10 dB. The second case involved positioning the elements on a common-ground plane. In this case, the antenna’s operational frequency is 4.81 GHz with an impedance bandwidth of 5.62% (4.67–4.94 GHz), a gain of 1.56 dBi, and a radiation efficiency of 58%. The reflection coefficient at the operating frequency was − 14.86 dB. The optimal spacing between the modules of antenna results in less inter-element coupling due to which the isolation is greater than 17.38 dB. The ECC was below 0.03, and the diversity gain was like that in Case 1. However, in both cases, the MIMO antenna design exhibited low gain, impedance bandwidth, and efficiency, leading to reduced performance. In^16^, a 2 × 2 MIMO framework was proposed, consisting of two semi-circular antenna components, a U-shaped stub, and a 50 Ω microstrip feedline. The suggested MIMO antenna design’s measurements are 16 × 21 × 1.6 mm^3^, and it uses an FR-4 substrate. A conductor strip was employed as a neutralizing line to reduce coupling among the ports. The MIMO antenna functions at 5.5 GHz, with a return loss of − 39.5 dB, gain of 2.8 dBi and isolation greater than 25 dB. The impedance bandwidth is 0.7 GHz (5.10–5.80) GHz, the ECC value is below 0.001, the mean effective gain (MEG) is – 6 dB, and the radiation efficiency is 84%. This antenna array is suitable for 5G data transmissions. However, the architecture has a narrow bandwidth, low gain and a complex structure owing to the use of a decoupling structure to enhance isolation. In^17^, a 2 × 2 MIMO antenna is suggested with measurements of 90 × 90 × 1.57 mm^3^. Since it is a dual band antenna, so it resonates at 3.56 GHz and 5.28 GHz with return loss of – 18 dB and − 23 dB, impedance bandwidth of 0.1 GHz (3.5–3.6) GHz and 0.17 GHz (5.21–5.38) GHz and gain is 4.2 dBi and 2.8 dBi. The isolation between MIMO components is greater than 22 dB, ECC value is 0.04 and DG is almost 10 dB. Despite its high gain, it has quite low bandwidth with large size. In^18^, 2-element MIMO Dielectric Resonator Antenna (DRA) with an inverted meandering U-shaped line engraved on it, is examined with measurements of 50 × 25 × 1.6 mm^3^. Due to its dual band design, it resonates at 3.7 GHz and 5 GHz with – 35 dB and – 21 dB, impedance bandwidth of 0.92 GHz (3.40–4.32) GHz and 0.09 GHz (4.96–5.05) GHz and gain is 12.87 dBi. Considering the MIMO diversity parameters, the isolation is greater than 22 dB, ECC is below 0.175 and MEG ratio is 0 dB. Despite its intriguing features, the bandwidth is quite narrow. The literature survey mentioned above shows that there are several constraints related to the bandwidth, gain, complexity of the structure, and diversity parameters. Therefore, it is essential to have a compact MIMO antenna design with significant gain, isolation, and broad frequency coverage in the sub-6 GHz frequency range of 5G technology.

Considering the limitations of antenna gain and design complexities for the sub-6GHz band, as per literature survey, we propose a MIMO antenna with four elements that offers significant and unique features for wireless communication in 5G sub-6 GHz networks. Furthermore, the proposed design exhibited increased segregation between the antenna components of the MIMO system and excellent radiation efficiency. By utilizing the proposed single antenna, an impressively performing four-element MIMO antenna was created, with each antenna element placed in an inline layout. This design stands out for its simplicity, broad operating frequency range (3.4–5.6 GHz), high gain of 3.01 dBi, and 71% radiation efficiency, along with significant isolation of over 20 dB among the MIMO antenna components. The proposed antenna designs were simulated using the 3D electromagnetic software CST Studio Suite 2019^19^ with its achieved numerical results cross-verified via another commercially available EM tool i.e, HFSS^20^ and later validated through experimentations.

Our suggested design offers the following contributions:

- Development of a quad-element MIMO antenna array with high gain, wide impedance bandwidth, and strong isolation for sub-6 GHz applications.
- A wideband characteristic bandwidth between 3.7 and 5.7 GHz and a gain of 1.81 dBi are achieved by the antenna design for the single-element configuration.
- The four-element MIMO architecture maintains a small size and broadband characteristics while delivering a realized gain of 3.01 dBi and radiation efficiency of 71% in the 5G range below 6 GHz.
- CMA antenna development tool was used in the design stages of antenna based on surface current modes and their distributions.
- Introduction of a vertical strip in the ground plane of the MIMO antenna design achieved a high isolation of − 38.53 dB and a gain enhancement of 3.01 dBi.

## Single element

The description of the configuration and blueprint approach for the single antenna element is described and thoroughly covered in this section. The computed results are also thoroughly examined including bandwidth, gain and radiation efficiency.

### Antenna layout

**Figure 2:**
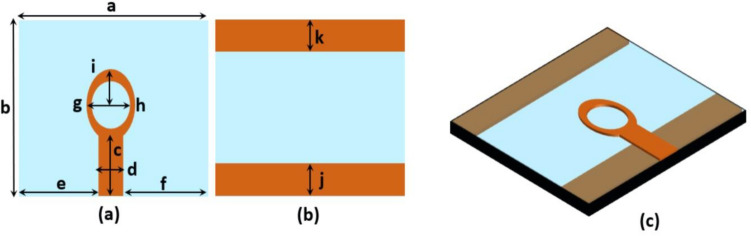
Single antenna element (**a**) Front view, (**b**) Rear view, (**c**) Perspective view.

The suggested single antenna element has a resonant frequency of 4.33 GHz with a bandwidth of 2 GHz (3.7–5.7 GHz). The design is composed of an elliptical patch with a circular slot, installed on low-cost FR-4 epoxy substrate of 1.6 mm thickness having permittivity and loss tangent of ${\varepsilon }_{r}$ = 4.4 and $tan\delta$ = 0.02. The antenna is energized by a 50 $\Omega$ microstrip feedline. On the back of substrate, two partial ground planes are created. The antenna measures 22 × 22 × 1.6 mm^3^*.* Figure 2 depicts the antenna’s optimum measurements and architecture. Copper is depicted by orange and substrate by blue color. The structural parameters were adjusted using the 3D-CST simulator to obtain antenna characteristics such as wide bandwidth, high gain and efficiency. The HFSS was also used to verify the design. The HFSS is based on finite element method, while the CST simulator is based on finite integration method. Table 1 depicts the final optimized geometrical dimensions.

**Table 1:** Optimized geometrical dimensions of the single element design.

Dimensions	Value (mm)
a	22
b	22
c	7
d	3.08
e	9.46
f	9.46
g	3.5
h	3.1
i	5.5
j	6.07
k	6.07

### Evolution phases of the design

**Figure 3:**
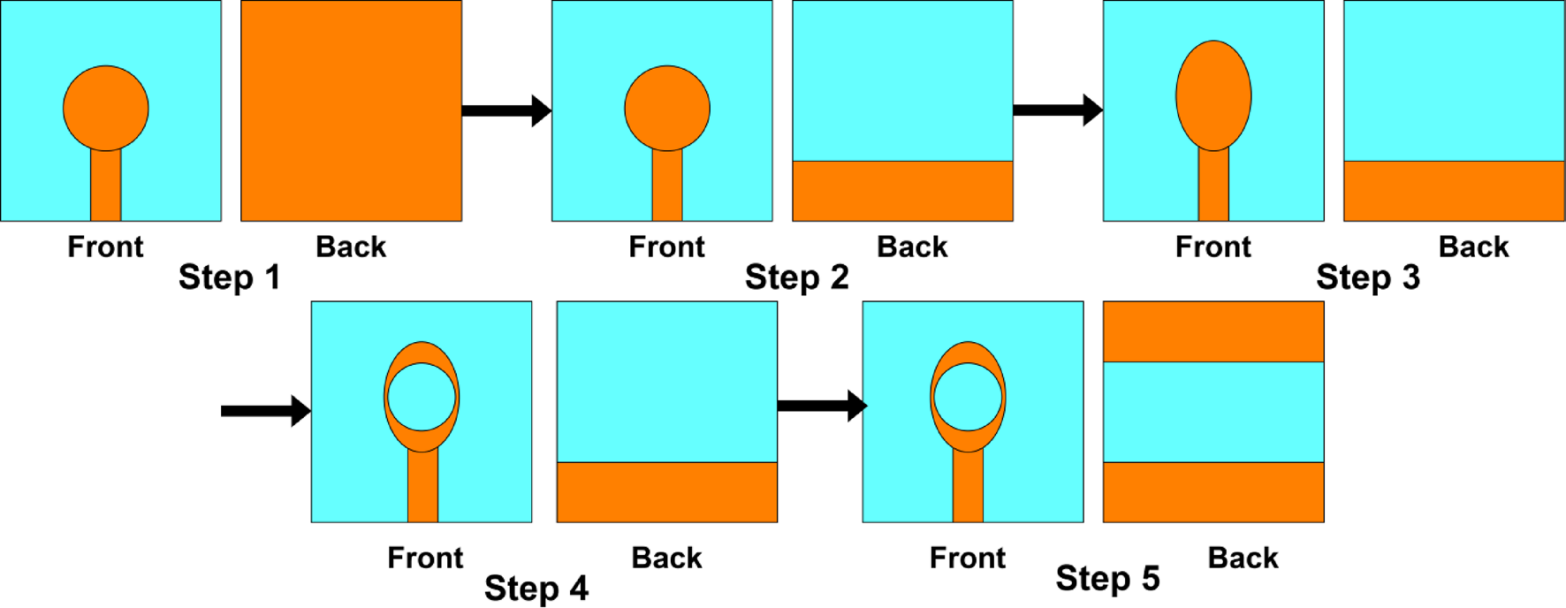
Evolution phases of single antenna element design.

The patch and the ground plane modification were performed to achieve a broadband antenna, enhanced gain and high efficiency. The evolution phases of the one-piece design are shown in Fig. 3. At the first step, a microstrip antenna based on circular patch with a full ground plane is energized by a 50 $\Omega$ microstrip feedline. In step 2, the ground plane is cut and turned into a segmented ground plane. The energy absorption in substrate is significantly reduced by a partial ground plane, and reduced energy conservation in substrate reduces the quality factor, so the bandwidth expands as the Q-factor drops. In step 3, the resonator with an elliptical patch replaces the circular one with a segmented ground plane to improve the antenna’s surface current route, causing the shifting of resonant frequency to a lower frequency range. In step 4, a circular slot is etched within the elliptical patch with a partial ground plane at the back. The fifth and the final stage involves creating two partial ground planes on the lower side with an elliptical patch having a circular slot on the top of the substrate.

**Figure 4:**
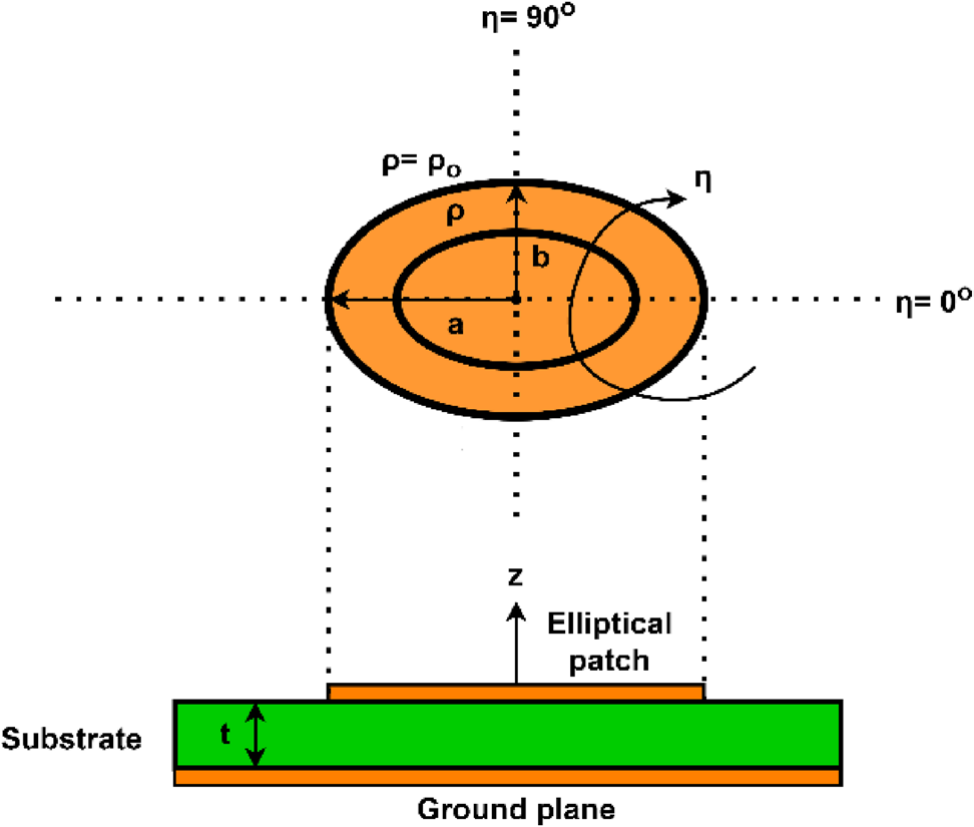
Elliptical-shaped patch with elliptical coordinates.

For calculating the elliptical-shaped patch antenna’s resonant frequency theoretically, we consider an elliptical patch resonator with major axis $a$ and minor axis $b$ carved on a dielectric substrate with relative permittivity of ${\varepsilon }_{r}$ and thickness of $t$ as shown in Fig. 4. The elliptical coordinate system is given as $\left(\rho ,\eta ,z\right)$. It is resonable to imagine that the elliptical patch has solely magnetic walls enclosing the sides and an electric wall enclosing the top and bottom^21^.

The two-dimensional wave equation in terms of cylindrical coordinates for elliptical patch is given as^22,23^:

1 $$\frac{{\partial }^{2}{E}_{z}}{\partial {\rho }^{2}}+ \frac{{\partial }^{2}{E}_{z}}{\partial {\eta }^{2}}+2q\left(cosh2\rho -cos2\eta \right){E}_{z}=0$$

where $2q=\omega h\sqrt{\mu \varepsilon }$ and $h$ denotes the ellipse given as:

2 $$\frac{{x}^{2}}{cos{h}^{2}\rho }+\frac{{y}^{2}}{sin{h}^{2}\rho }={h}^{2}$$

Solving the equation of $h$, we get $h=ae$, where $e$ is the eccentricity ${e}^{2}=1-{\left(\frac{b}{a}\right)}^{2}$. Wave equation in Eq. (1) has solution given as, neglecting the time dependence element, ${e}^{j\omega t}$:

3 $${E}_{z}=\sum_{m=0}^{\infty }[{C}_{m}C{e}_{m}\left(\rho ,q\right) C{e}_{m}(\eta ,q)]+\sum_{m=1}^{\infty }[{S}_{m}S{e}_{m}\left(\rho ,q\right) S{e}_{m}(\eta ,q)]$$

Equation (3) indicates that there are two different and separate kind of modes where $C{e}_{m}\left(\rho ,q\right) C{e}_{m}\left(\eta ,q\right)$ corresponds to even modes while $S{e}_{m}\left(\rho ,q\right) S{e}_{m}\left(\eta ,q\right)$ corresponds to odd modes.

Using Maxwell’s equations, other fields are calculated as:

4 $${H}_{\rho }=\frac{j}{\omega \mu ae(cos{h}^{2}\rho -{cos}^{2}\eta {)}^\frac{1}{2}}\frac{\partial {E}_{z}}{\partial \eta }$$

5 $${H}_{\eta }=\frac{-j}{\omega \mu ae(cos{h}^{2}\rho -{cos}^{2}\eta {)}^\frac{1}{2}}\frac{\partial {E}_{z}}{\partial \rho }$$

To calculate resonant frequency for elliptical patch, the following boundary condition must be satisfied.

6 $${H}_{\eta }=\frac{\partial {E}_{z}}{\partial \rho }{|}_{\rho ={\rho }_{o}}=0$$

where $\rho ={\rho }_{o}$ denotes the boundary of ellipse. Thus, the resonance conditions will be:

7a $$C{e}_{m}{\prime}\left({\rho }_{o},q\right)=0\; For\, even\, or\, {TM}_{cmn}\, modes$$

7b $$S{e}_{m}{\prime}\left({\rho }_{o},q\right)=0 \;For\, odd\, or\, {TM}_{smn}\, modes$$

here the prime indicates the derivative, the first subscript $c or s$ indicates the Mathieu function’s type (even or odd), the second subscript $m$ denotes the order of Mathieu function and its ${n} {\text{th}}$ zero is indicated by the last subscript $n$.When the eccentricity approaches zero, $Eq.$ (7) approaches to a circular solution of ${J}_{n}{\prime}\left(kr\right)=0$ where ${J}_{n}\left(kr\right)$ is the Bessel function of order $n$. Solving Eqs. (7a and 7b) yields

8 $$2\sqrt{{q}_{mn}=aek}$$

where $k=\omega \sqrt{\mu \varepsilon }$ is the propagation constant and ${q}_{mn}$ is the eigen value. The resonant frequency is given as

9 $${f}_{mn}=\frac{c\sqrt{{q}_{mn}}}{\pi ae\sqrt{{\varepsilon }_{r}}}$$

where $c$ is the speed of light.

Both the eigen values of TM modes and eigen values of TE modes in elliptical-shaped waveguide having identical measurements and eccentricity are equal. So, the formula calculated by Kretzschmar^22^ for ${TE}_{c01}, { TE}_{c11}, {TE}_{s11}, { TE}_{c21} and {TE}_{s21}$ are used for calculating the eigen values of corresponding TM modes in elliptical-shaped patch antenna.

The influence of fringing fields has not been factored into the resonant frequency calculation in Eq. (9). So, the Eq. (9) is modified to include the effect of fringing fields also^24,25^. Elliptical patch has area given as $S=\pi ab$. ${a}_{eq}$ shows the radius of a circular patch that is comparable to an elliptical patch having identical area. So,

10a $$S=\pi {a}_{eq}^{2}=\pi ab$$

10b $${a}_{eq}=\sqrt{ab}$$

Effective radius of circular patch is calculated as

11 $${a}_{eff}={{a}_{eq}\left[1+\frac{2t}{{a}_{eq}{\varepsilon }_{r}}\left(ln\frac{\pi {a}_{eq}}{2t}\right)+1.7726\right]}^{1/2}$$

If we replace $a$ by ${a}_{eff}$ in Eq. (9), the modified resonant frequency for elliptical patch is given as

12 $${f}_{mn}=\frac{c\sqrt{{q}_{mn}}}{\pi {a}_{eff}e\sqrt{{\varepsilon }_{r}}}$$

**Figure 5:**
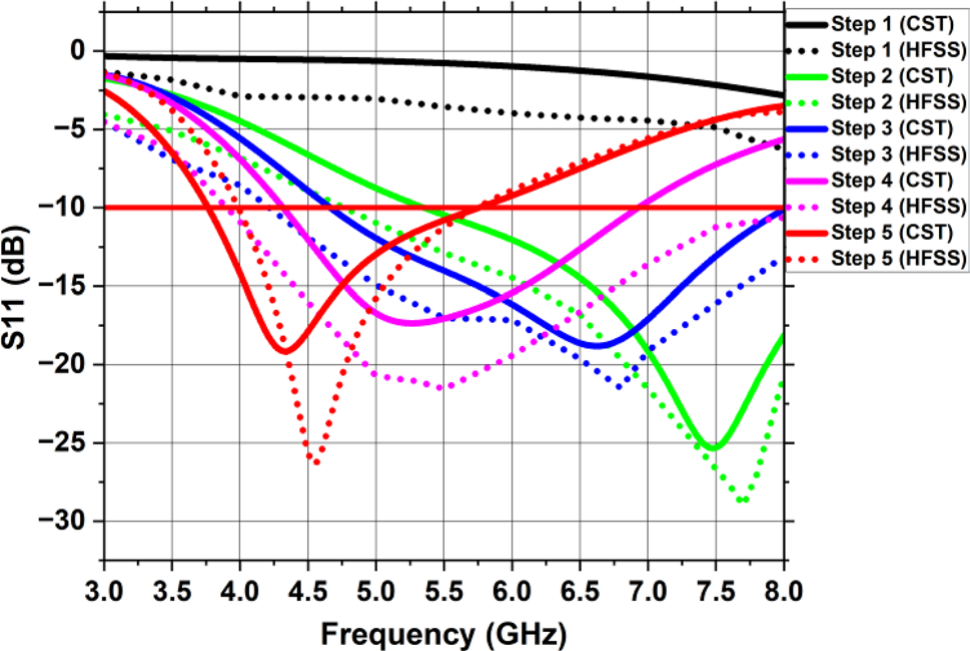
Reflection coefficient of each stage of single antenna element using CST and HFSS.

The simulated reflection coefficient (S11) for the steps mentioned earlier is shown in Fig. 5. For step 1, the designed antenna fails to achieve any – 10 dB working band due to impedance imbalance between the typical patch and the full ground plane. In step 2, the antenna’s resonant frequency is 7.47 GHz with an impedance bandwidth of 3.8 GHz (5.3–9.1 GHz) and gain is 0.875 dBi. In the third step, the antenna resonates at 6.625 GHz with an impedance bandwidth of 3.34 GHz (4.65–7.99 GHz) and gain is 1.278 dBi. This is due to the improvement in the antenna’s surface current route. In step 4, the etching of circular slot in the elliptical patch shifts the resonant frequency to 5.26 GHz with an impedance bandwidth of 2.6 GHz (4.3–6.9 GHz) and gain is 1.726 dBi. This is due to the elongation of surface current path. The final step involves creating two partial ground planes on the bottom of substrate with an elliptical patch having circular slot on the top which allows the antenna to accomplish a resonant frequency of 4.33 GHz with an impedance bandwidth of 2 GHz (3.7–5.7 GHz) as well as a gain of 1.81 dBi. The current path was lengthier in the patch and spread over a greater distance than in the previous stages. These alterations in the structure consequently contribute to the enhancement of gain, the reflection coefficient, and the radiation directivity.

### Parametric analysis

Parametric optimization and analysis were performed to identify the influence of the slot and various dimensions of patch and ground on the ultimate outcomes of the proposed antenna. The values of the various parameters were adjusted using parametric analysis. These parameters control the resonant characteristics and bandwidth of the design. This subsection presents several parameters that influence the antenna’s overall performance.

### Analysis of different width of ground plane *(j/k)*

**Figure 6:**
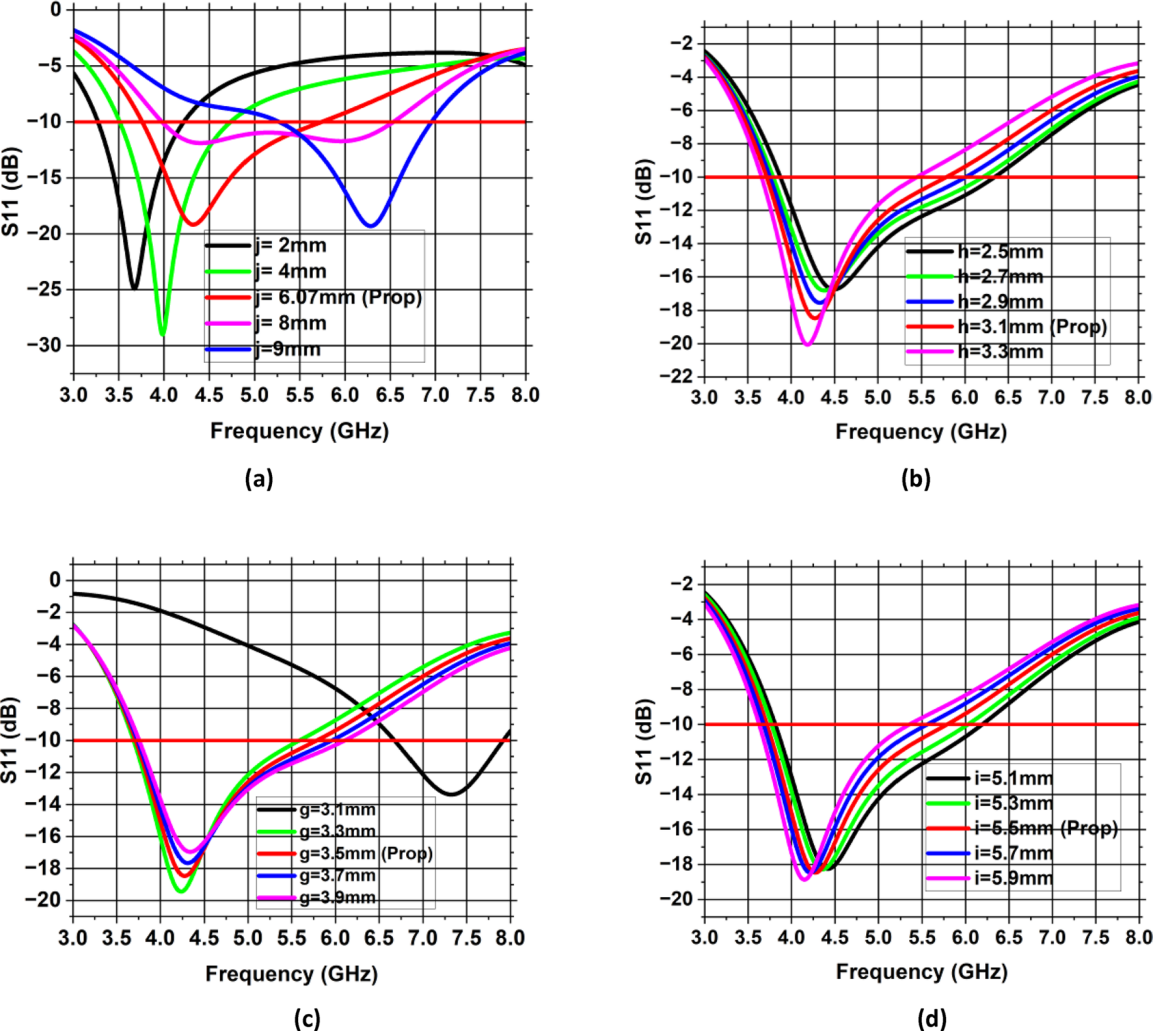
Parametric analysis of single antenna element (**a**) width of ground plane *j * or *k*, (**b**) radius of circular slot *h*, (**c**) minor axis of elliptical patch *g*, (**d**) major axis of elliptical patch *i*.

A parametric analysis of various width values j or k was conducted to obtain desired resonance frequency with appropriate impedance matching. This analysis is mainly utilized to enhance single antenna performance. In the parametric study, the width value was varied from 2 to 9 mm with increment of 2 mm. The study revealed that the dimensions under consideration significantly impacted the antenna’s performance, with *j* = *k* = 6.07 mm producing the desired outcomes. The simulated S11 is presented in Fig. 6a for various values of *j* or *k*, where the desired resonance frequency with optimal impedance bandwidth is seen at *j* = *k* = 6.07 mm*.*

### Analysis of radius of circular slot (*h*)

As shown in Fig. 6b, a parametric study of radius of circular slot *(h)* was undertaken to analyze its influence on the antenna performance. The radius of slot is varied from 2.5 to 3.3 mm with increment of 0.2 mm. Etching of circular slot results in the introduction of capacitance in the antenna design. As the radius of slot increases, the capacitance also increases which results in the shifting of resonant frequency to lower side. With optimum value of h = 3.1 mm, the suggested antenna resonates at a desired frequency of 4.33 GHz with a bandwidth of 2 GHz (3.7–5.7 GHz).

### Analysis of minor axis of elliptical patch (*g*)

Another parameter is the minor axis of elliptical patch which appears to have an impact on the desired frequency and reflection coefficient. Figure 6c illustrates the impact of minor axis of elliptical patch on antenna performance. As the value of *g* is increased, with all other parameters remaining constant, the inductance increases which results in the shifting of resonance frequency to lower side. This parametric investigation was carried out by changing the minor axis of the elliptical patch g from 3.1 to 3.9 mm with increment of 0.2 mm. Figure 6c shows that the desired resonance frequency with optimum impedance matching are observed at g = 3.5 mm*.*

### Analysis of major axis of elliptical patch (*i*)

The impact of the major axis of the elliptical patch on the antenna’s resonant frequency and input impedance matching is depicted in Fig. 6d. The analysis was conducted with varying elliptical patch’s major axis (*i*), ranging from 5.1 to 5.9 mm in increments of 0.2 mm. Figure 6d shows the simulated S11 for various major axis, wherein it can be seen that elliptical patch’s major axis has an impact on antenna’s resonant frequency. Value of *i* = 5.5 mm provides the desired operating frequency of 4.33 GHz with optimum value of reflection coefficient.

### Characteristic mode analysis (CMA)

The CMA approach is utilized for examining the design behavior in terms of electromagnetic modes by applying it to the five stages of the individual antenna element that is being suggested is depicted in Fig. 3. In this analysis, the focus is solely on the conducting element i.e. the top later elliptical slot patch and the ground plane of the antenna, with no consideration given to the substrate. As the CMA does not incorporate substrate material, the dielectric constant is assumed to be ${\varepsilon }_{r}=1$, corresponding to air. CMA stands as a robust method for the analysis and design of antennas, employing a mathematical approach to ascertain the radiation parameters of an antenna by dissecting it into its basic modes. The core idea of CMA involves illustrating the electromagnetic fields of an antenna as a linear mixture of its distinctive modes—those capable of independent radiation. This enables a comprehensive understanding of the antenna’s underlying features and facilitates performance optimization through breaking down the radiation into these unique modes. To ascertain the distinctive modes of the antenna, the CMA technique involves solving an eigenvalue constraint. The frequencies where the antenna resonates effectively are represented by the eigenvalues. Conversely, the eigenvectors delineate the modes and polarization of the radiation pattern.

Robert J. Garbacz led the investigation on the fundamental concept of CMA in 1965, introducing the “the occurrence of modal extension on resonating area in electromagnetic dispersion"^26,27^. A foundational concept of CMA involves the weighted aggregate of $N$ perpendicular eigencurrents can be used to represent the entire current brought forth by an advancing electromagnetic field going via a structure that conducts electricity or a framework that emits $({J}_{i})$. These eigencurrents depend on both the excitation vector and the geometry and material of the structure^28,29^. Equation (13) shows the entire current, and the influence of eigencurrents on the whole current are determined by modal coefficients of weight $(i)$. Since each eigencurrent produces its own unique impact, the whole current helps to create the emitted electric field.

13 $$J=\sum_{i=N}^{N}{\beta }_{i}{J}_{i}$$

Modal significance (MS) determines the highest normalized current magnitude for every mode. It is a crucial CMA factor which influences the radiation characteristics of that specific mode. Eq. (14) can be employed to calculate the modal significance.

14 $$MS=\left|\frac{1}{1+{j}_{i}{\lambda }_{i}}\right|$$

MS is inversely related to the eigenvalue $({\lambda }_{i})$ in Eq. (14). Equations (15) and (16) explain a way of moment matrix $([Z])$ along with an eigencurrent that can be utilized to calculate the $\left({\lambda }_{i}\right).$

15 $$\left[Z\right]=\left[R\right]+j\left[X\right]$$

16 $$\left[X\right]{J}_{i}={\lambda }_{i}\left[R\right]{J}_{i}$$

$R$ and $X$ define the real and imaginary components of $Z$. As seen in Table 2, the eigenvalues are significant because they can be used to forecast the inside energy or resonance in conducting materials. Utilizing the characteristic angle (CA) allows for the explanation of the electric field and surface current’s phase difference of the antenna. Equation (17) serves as a tool to calculate the characteristic angle (CA) represented by ${\alpha }_{i}$^30^.

**Table 2:** Important CMA characteristics.

Eigenvalues ${\lambda }_{i}$	Characteristic angle ${\alpha }_{i}$	Importance
${\lambda }_{i}<0$	${{180}^{\circ}<\alpha }_{i}<{270}^{\circ}$	Electric energy
${\lambda }_{i}=0$	${\alpha }_{i}={0}^{\circ}$	Resonating point
${\lambda }_{i}>0$	${{90}^{\circ}<\alpha }_{i}<{180}^{\circ}$	Magnetic energy

17 $${\alpha }_{i}={180}^{\circ}-{tan}^{-1}{\lambda }_{i}$$

**Figure 7:**
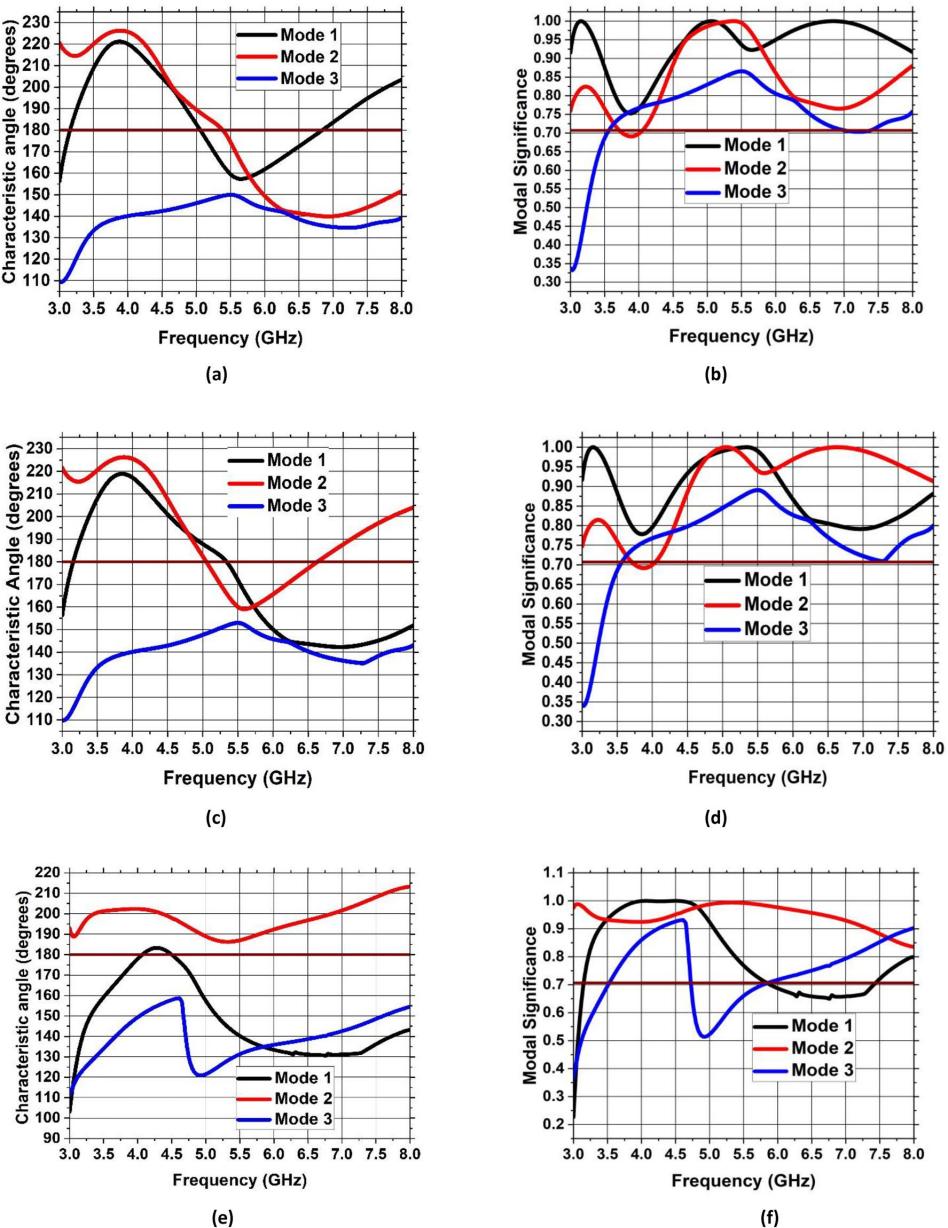
CMA analysis of evolution phase of single antenna element (**a**) CA of Step 1, (**b**) MS of Step 1, (**c**) CA of Step 2, (**d**) MS of Step 2, (**e**) CA of Step 3, (**f**) MS of Step 3.

**Figure 8:**
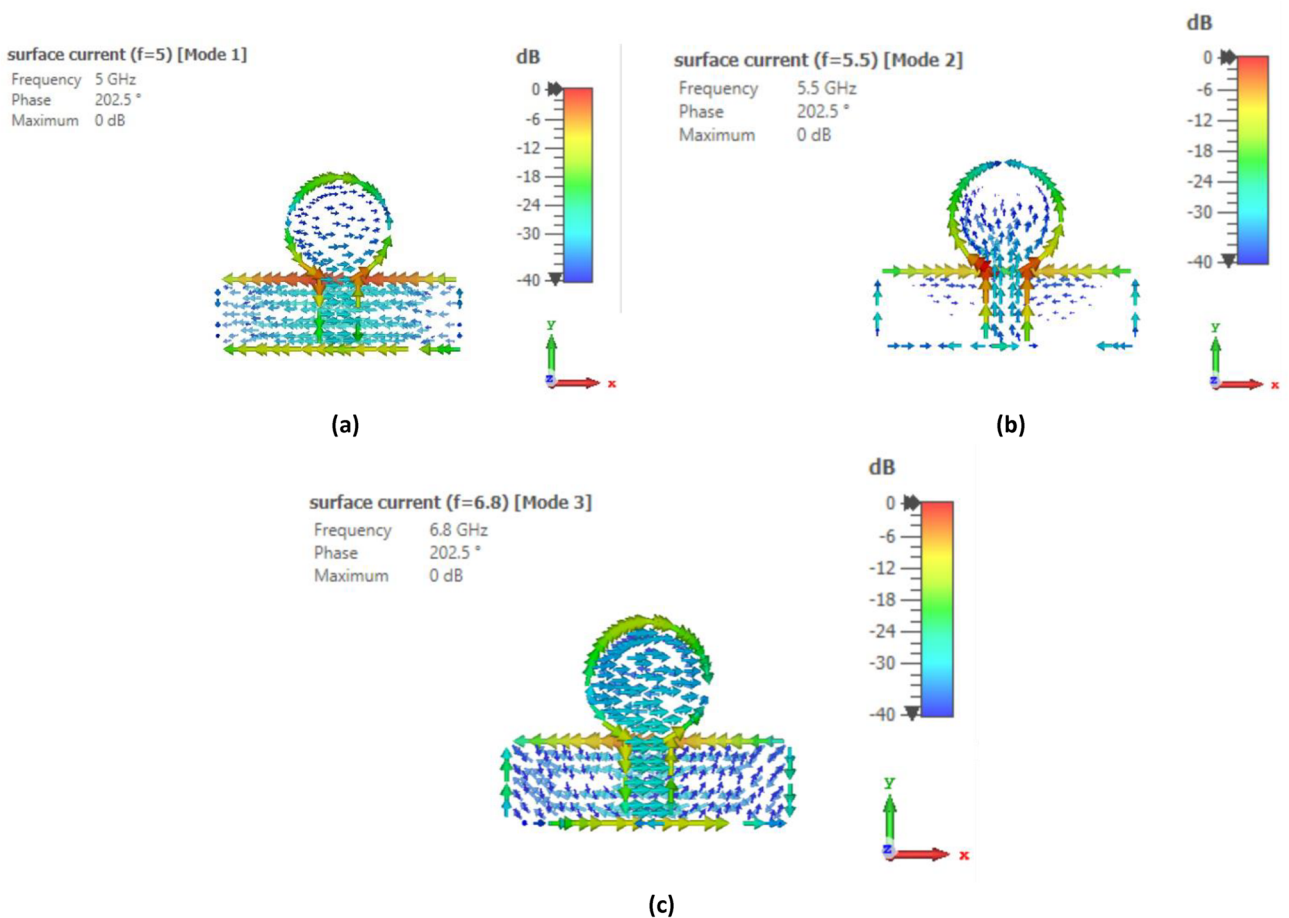
Surface current distribution of Step 1 at (**a**) First mode at 5 GHz, (**b**) Second mode at 5.5 GHz, (**c**) Third mode at 6.8 GHz.

**Figure 9:**
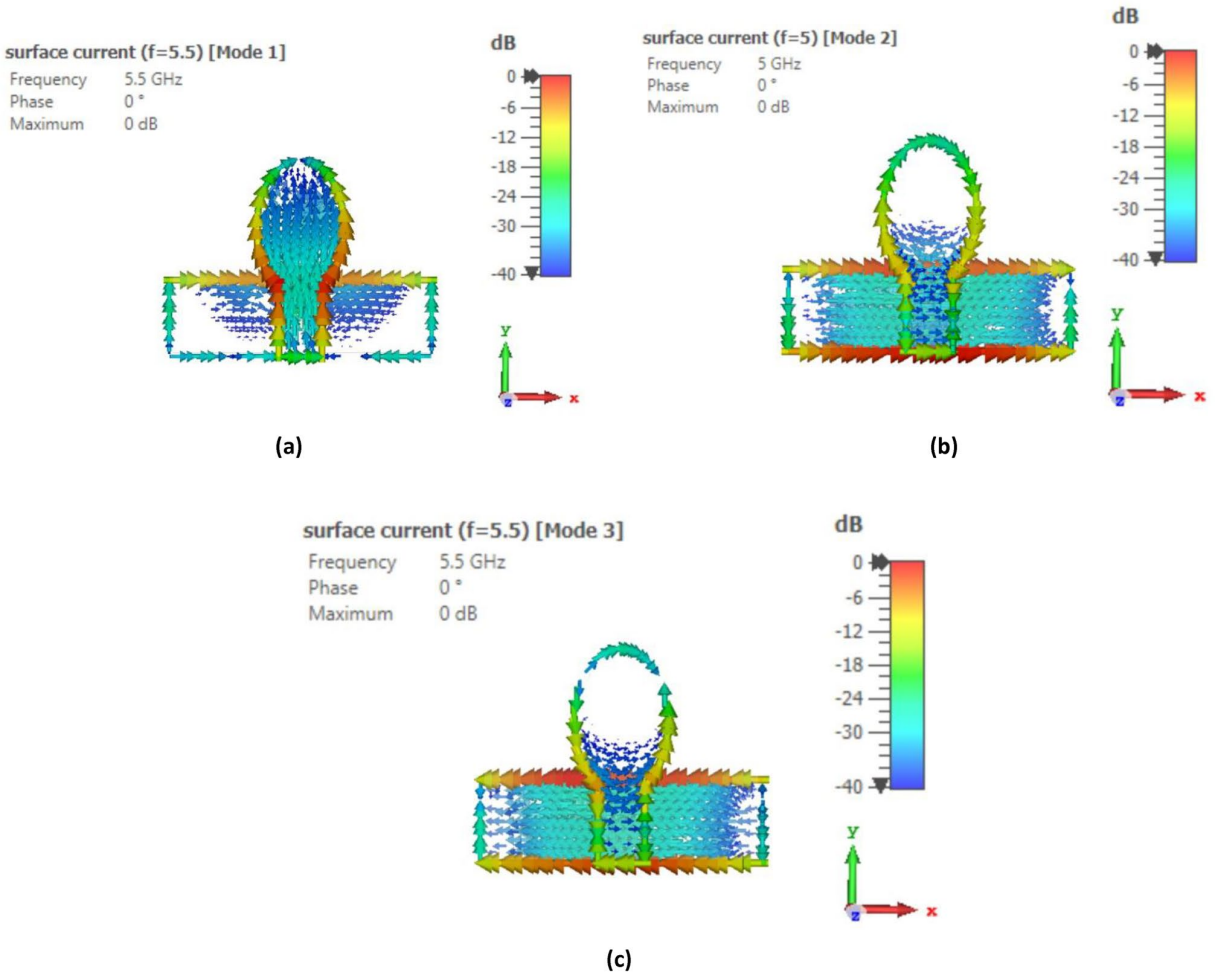
Surface current distribution of Step 2 at (**a**) First mode at 5.5 GHz, (**b**) Second mode at 5 GHz, (**c**) Third mode at 5.5 GHz.

**Figure 10:**
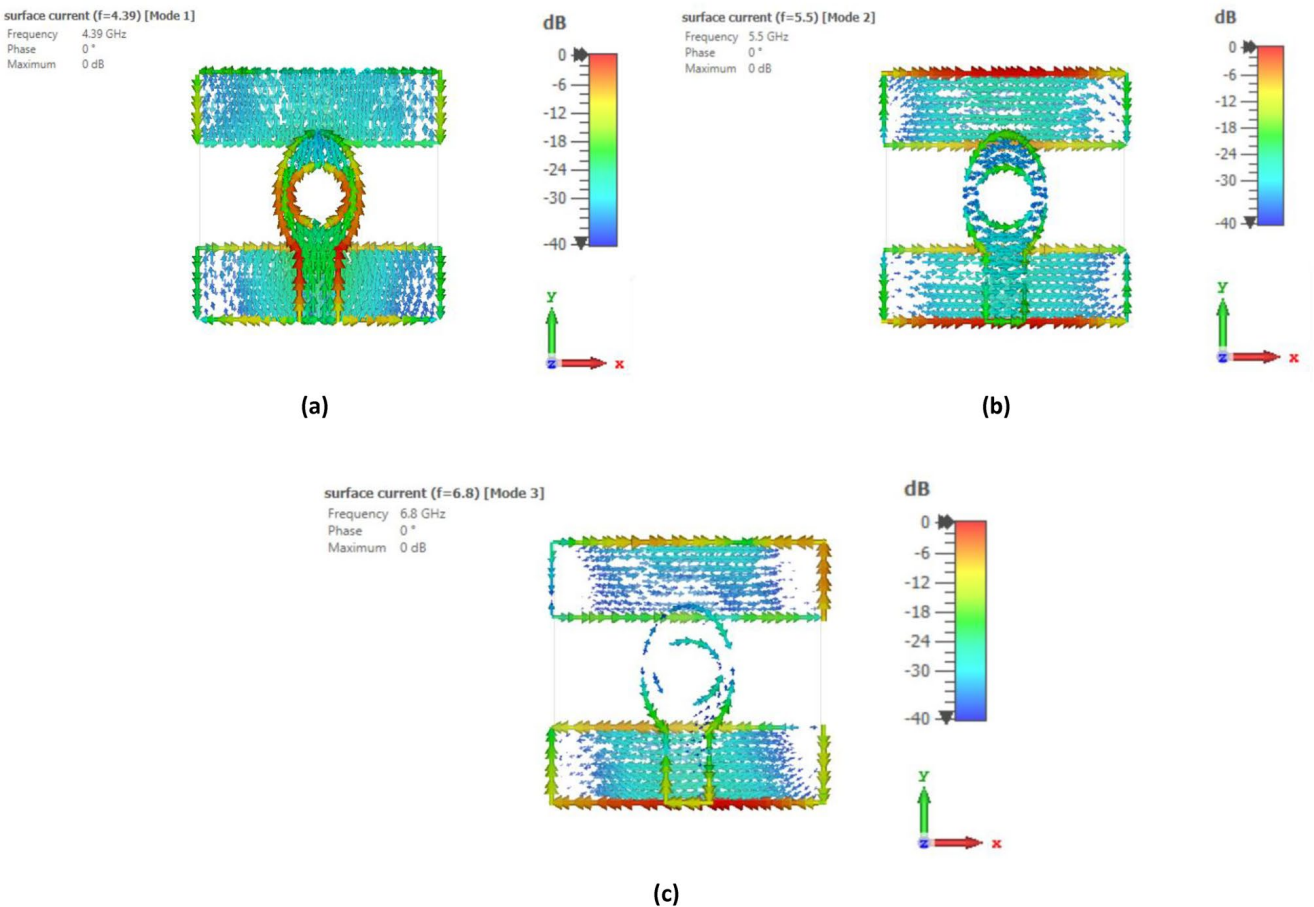
Surface current distribution of Step 3 at (**a**) First mode at 4.3 GHz, (**b**) Second mode at 5.5 GHz, (**c**) Third mode at 6.8 GHz.

To identify the noteworthy modes along with their impact on the radiation of antenna, the recommended antenna energized by single port is examined in detail using Characteristic Mode Analysis (CMA). Figure 7 presents the Modal Significance (MS) and Characteristic Angle (CA) for the three modes of the antenna. A mode is deemed significant if its MS exceeds 0.707^31^. With reference to Fig. 3, the evolution phases based on five steps are presented and analyzed with the CA and MS graphs. The three modes are generated at the resonant frequency of 4.33 GHz. It can be seen from Fig. 7 that for step 2, first mode exhibits two resonances, seen at the CA value of ${180}^{\circ}$ at 5 GHz and 6.8 GHz accordingly while second mode exhibit resonance at 5.5 GHz. The third mode is having a CA value of below ${180}^{\circ}$, thus illustrating that it retains energy like magnetic energy. Same analysis can be seen for the MS values in the range of 0.7 and above. When moving to step 3 of the evolution design, the modes are defined as mode 1 having now only one resonance at 5.5 GHz while mode 2 is resonating at 5 GHz with mode 3 still storing magnetic energy. For the final step 5 of the evolution design, the resonance of first mode is only appearing at the CA value of ${180}^{\circ}$ with resonances in the 4–4.4 GHz range. Surface current distributions are also analyzed for the CMA modes to confirm the antenna design and its evolution. Figures 8, 9 and 10 show the surface current distribution according to the CA analysis. As illustrated in Fig. 8a and b step 2, the modes 1 and 2 have dominant fields around the circular patch edges and the ground plane (orange-yellowish arrows) while the weak currents are at the center of the both the conducting elements (blue arrows). For mode 3, most the energy is stored energy and is available at the center of the ground plane and circular patch as shown in Fig. 8c. To reach towards step 3, the circular shape was redesigned towards an elliptical one to concentrate the energy and then look for resonance at the desired band of interest i.e. 4.33 GHz. Figure 9 depicts the surface current behavior of step 2 where most of the energy is confined in the elliptical design. Finally step 5 introduced slot in the elliptical patch and an additional partial ground was added to shift the resonance towards the central frequency. Figure 10 depicts the surface current behavior of the final single element design where mode 1 resonates while mode 2 and mode 3 are stored magnetic energy.

### Results and discussion

**Figure 11:**
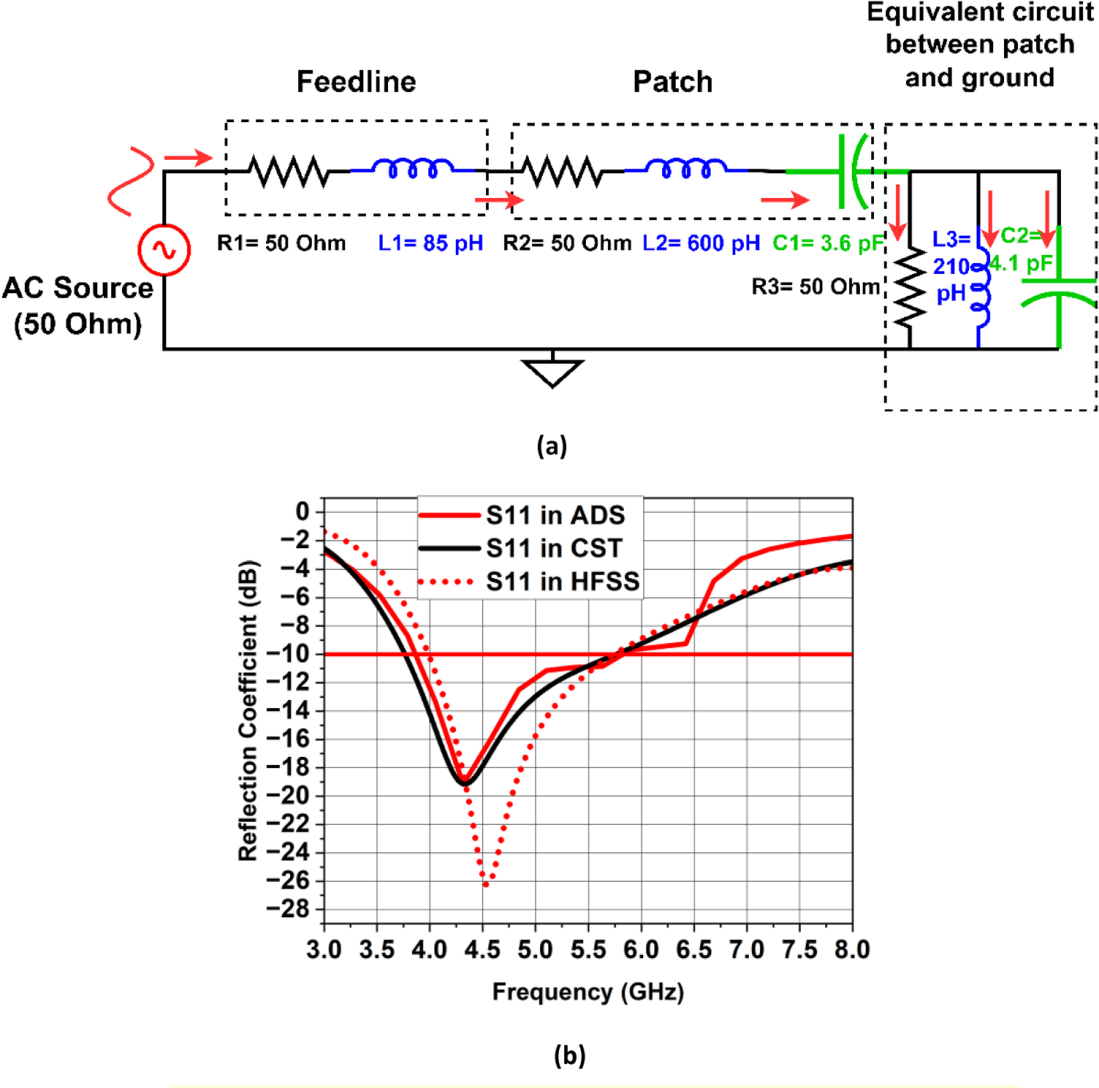
(**a**) Corresponding circuit design of the single antenna element, (**b**) Reflection coefficient simulated in CST vs HFSS vs ADS.

**Figure 12:**
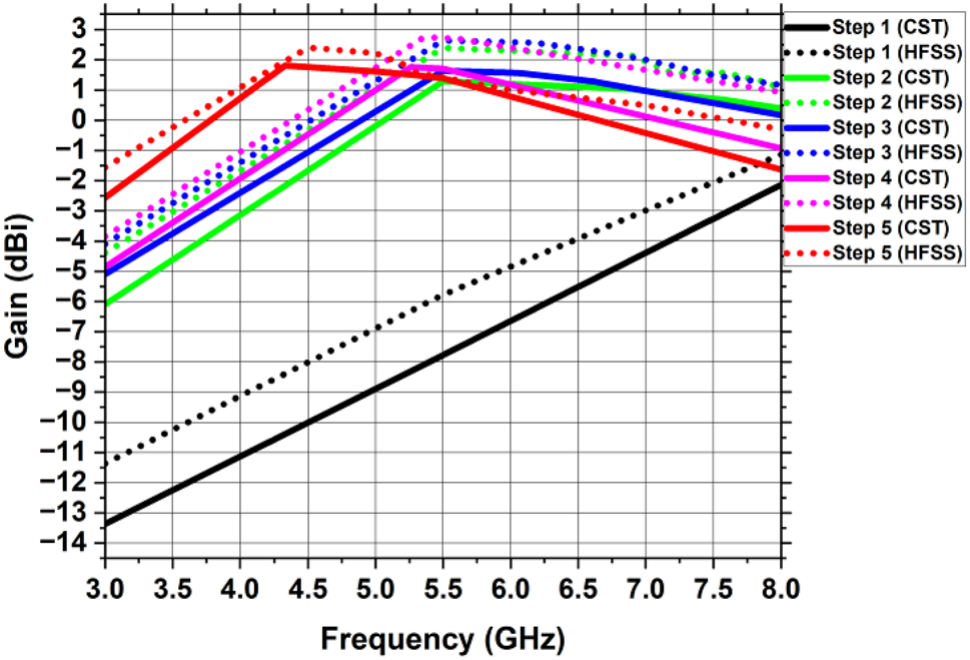
Gain of the single antenna element at every stage of the development.

**Figure 13:**
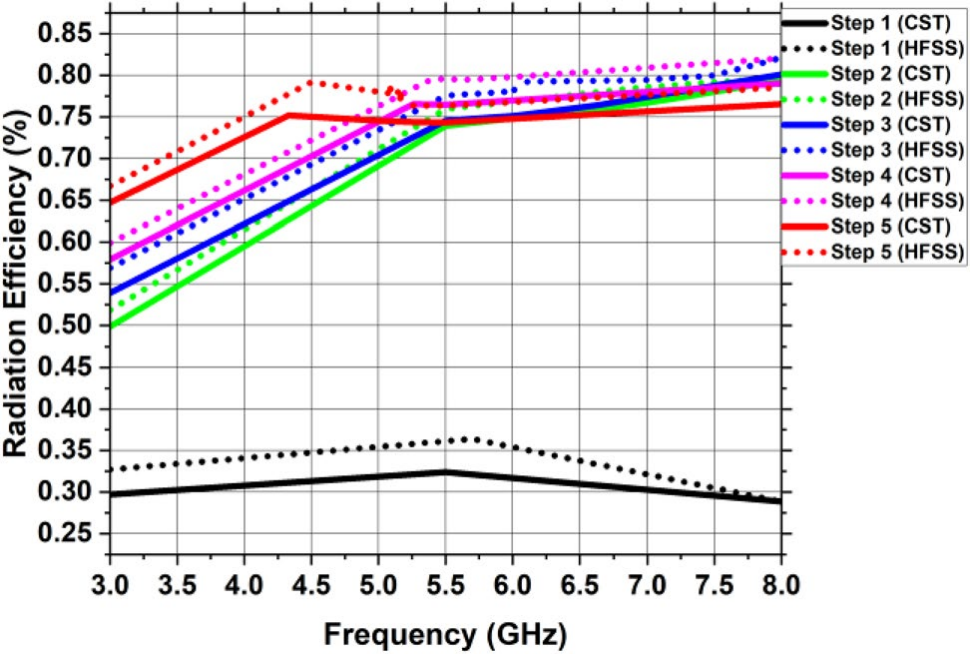
Radiation efficiency of the single antenna element at every stage of the development.

Figure 11(a) depicts the single antenna design’s corresponding circuit model. This circuit has been designed in Advanced Design System software (ADS)^32^. The concept of transmission line model is used which is based on designing circuit module using lumped elements which resonate at a specific resonant frequency. In this model, the series combination of R1 and L1 shows the comparable lumped model of the 50 $\Omega$ microstrip feedline. The elliptical-shaped patch is represented by series combination of $R2, L2$ and $C1$, where $C1$ is the capacitance that results from the circular slot. The analogous model between the elliptical-shaped patch with circular slot and partial ground plane is represented by the parallel combination of $R3, L3$ and $C2$. Some of the inductance caused by the fringing fields and surface waves is accounted for by the inductance $L3$. Figure 11b shows the comparison of S11 parameter simulated in ADS and CST software. Figures 12 and 13 depict the gain and radiation efficiency of the design at each step. It can be observed that the gain of the design is increasing at each step of evolution of antenna, from 0.875 to 1.81 dBi. This gain improvement is due to altering the shape of ground plane and patch antenna which results in the reduction of backward radiation and increases Front-to-Back Ratio (FBR). As a result, radiation efficiency has also increased.

**Figure 14:**
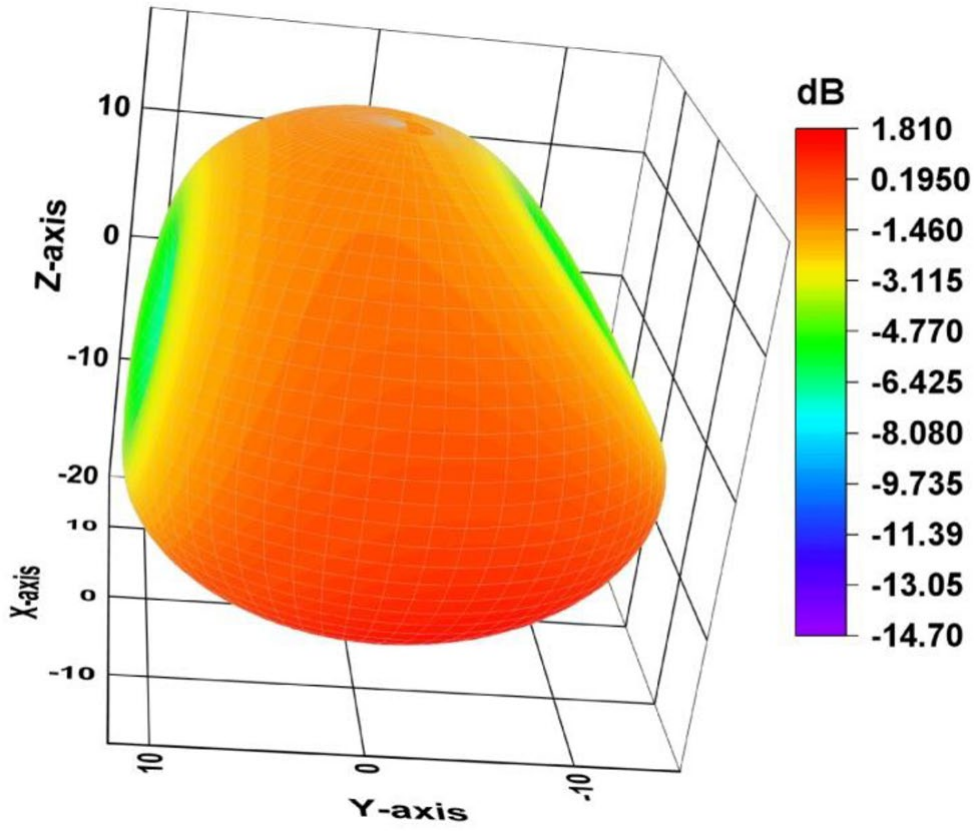
3D gain plot of the single antenna element.

Figure 14 shows a 3D gain figure of the suggested single antenna element design whose operating frequency is 4.33 GHz. It is omnidirectional in nature with an overall gain of 1.81 GHz.

## MIMO antenna design

When a MIMO transmits at the ${i} {\text{th}}$ node, its channel matrix is a $nL {\rm X} nM$ diagonal matrix, with the diagonal entries represented by $H[k{]}_{i}$, according to the antenna design paradigm indicated above. Using a complex zero-mean Gaussian random distribution, independent and identically distributed (i.i.d) random variables are used to describe the fading for the ${k} {\text{th}}$ OFDM sub-carrier, which is represented by the $H[k{]}_{i}$. The signal that the ${i} {\text{th}}$ node acquired is provided by,

18 $${y}_{i}={H}_{i}{x}_{i-1}+{n}_{i}$$

where ${n}_{k}$ is a $nL\times 1$ complex additive noise vector at the ${i} {\text{th}}$ node, characterized as an i.i.d zero-mean, circularly symmetric, complex Gaussian random vector with variance ${\sigma }_{k}^{2}$. The transmitted signal is represented by ${x}_{i-1}$, a vector of size $nM \times 1$^33^.

To attain MIMO antenna characteristics, a quad element design has been proposed. The elements are positioned in an inline arrangement on a FR-4 substrate whose measurements are 44 × 44 × 1.6 mm^3^. The suggested quad-element MIMO’s basic building block consists of four-elliptical shaped radiators with a circular slot in each radiator, limited ground plane coupled with a vertical strip and a microstrip feedline. The substrate used for its construction is FR-4 which has a dielectric strength of ${\varepsilon }_{r}$ = 4.4 and $tan\delta$ = 0.02. Each part works in tandem with the others and vertical strip placed in the ground plane improves matching. Two MIMO configurations are discussed in the subsequent sections.

### 2 × 2 MIMO antenna design with no vertical strip

**Figure 15:**
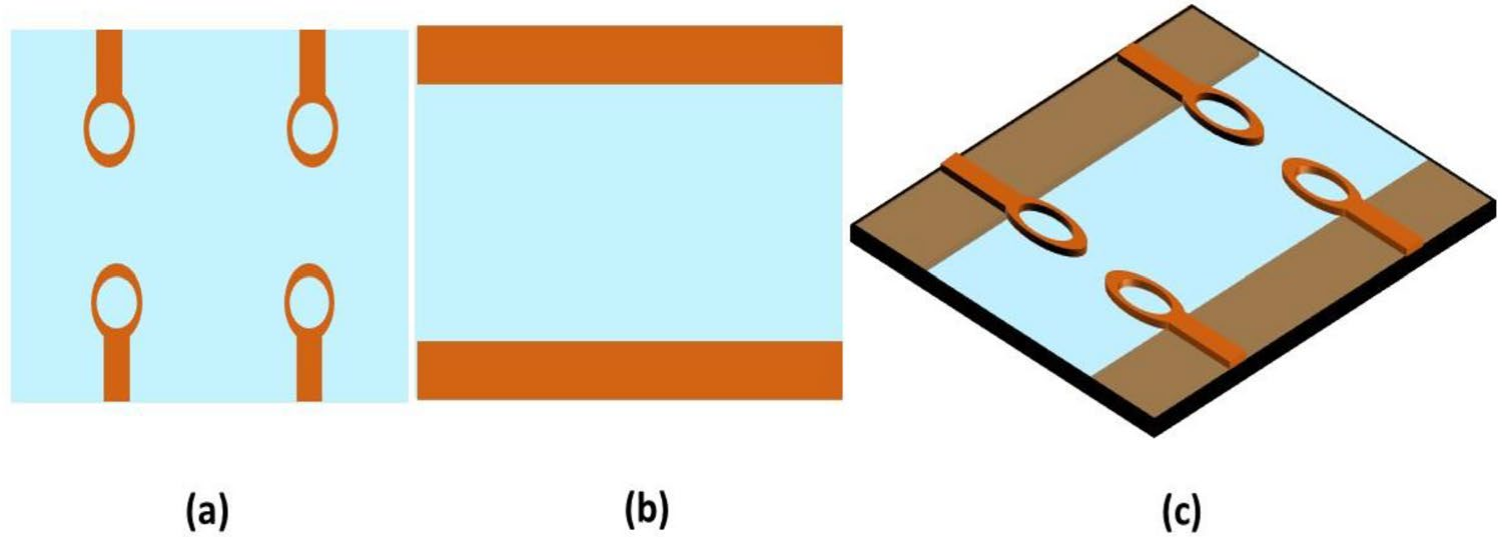
A 2 × 2 MIMO antenna without vertical strip (**a**) front view, (**b**) back view, (**c**) exploded view.

**Figure 16:**
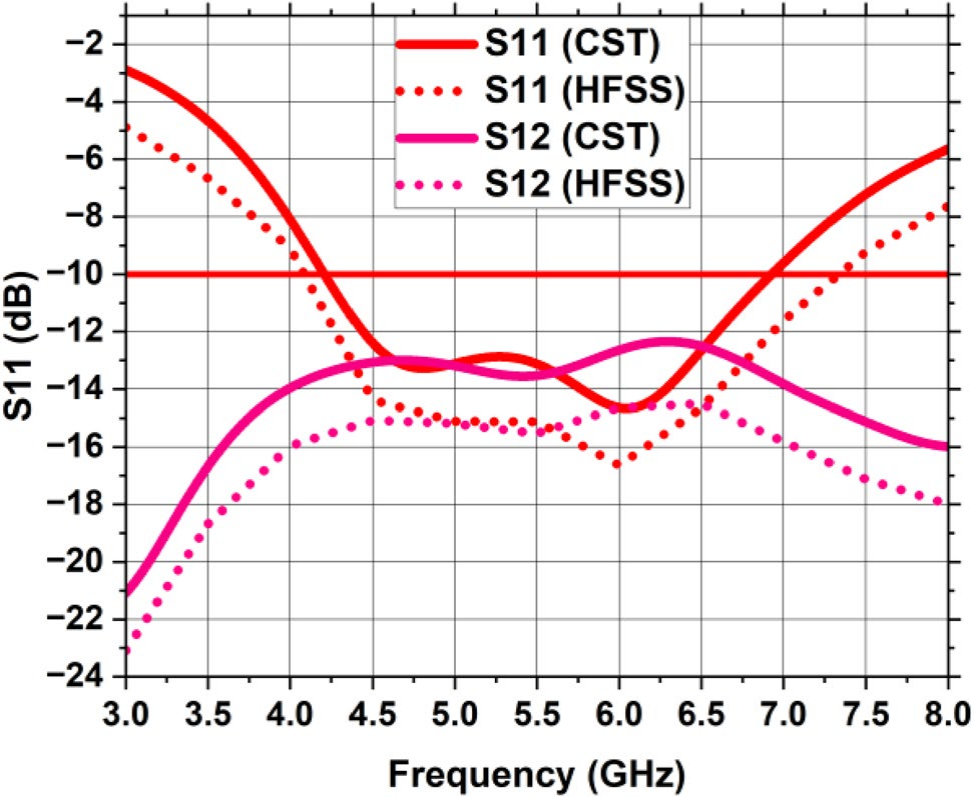
Reflection and Transmission coefficient of MIMO antenna without vertical strip.

Figure 15 depicts the view of the 2 × 2 MIMO setup from the front and rear side without any vertical strip in the ground plane. Figure 16 shows the S11 and S12 of the suggested design, as well as the fact that in the absence of a vertical strip, inter-element coupling is very high due to low isolation. This design has a bandwidth of (4.2–6.9) GHz with center frequency of 6 GHz. Due to mutual coupling between the antenna elements, there is a change in the impedance matching due to which it resonates at a frequency of 6 GHz. Vertical Strip in the partial ground plane is used to reduce the inter-element coupling and achieve the impedance matching at the desired operating frequency, since the length and width of vertical strip plays a vital role in changing the resonant frequency and bandwidth of MIMO antenna. The introduction of vertical strip results in the increase of capacitance effect of MIMO antenna due to which the resonant frequency decreases.

### 2 × 2 MIMO antenna design with a vertical strip

**Figure 17:**
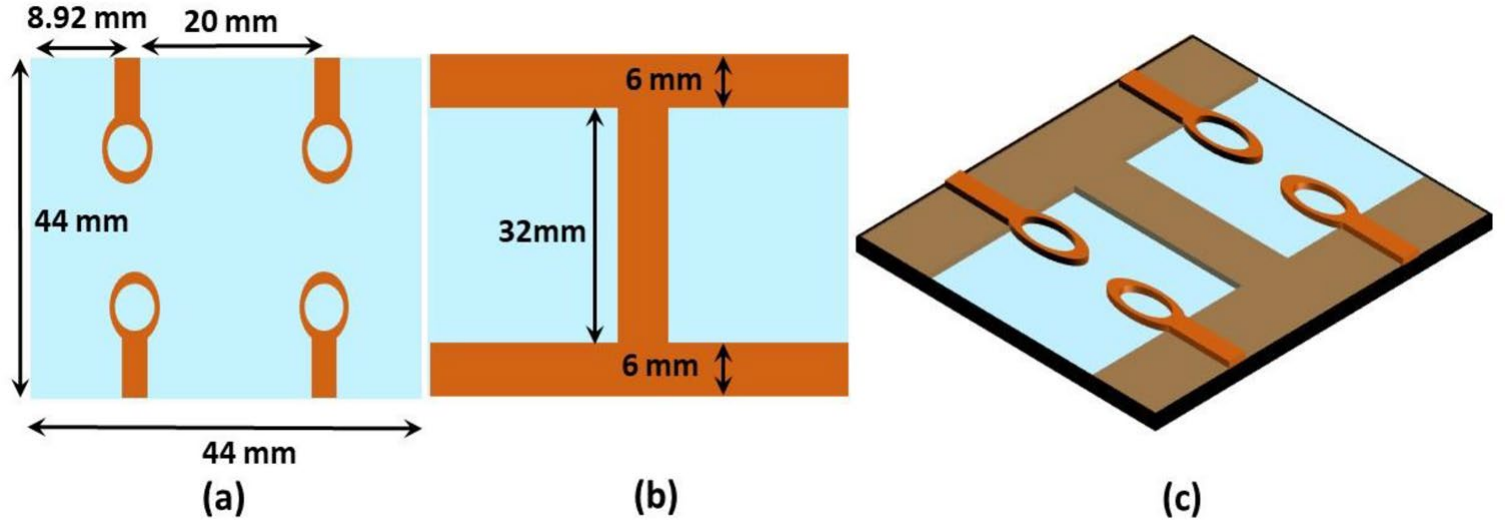
A 2 × 2 MIMO antenna including vertical strip (**a**) front view, (**b**) back view, (**c**) exploded view.

The 2 × 2 MIMO antenna layout comprising an imperfect ground plane including a vertical strip is depicted in Fig. 17 with measurements mentioned for each radiating component. The inclusion of the vertical strip has established appropriate isolation between MIMO components. The simulated and measured reflection coefficients (S11, S22, S33, and S44) of the

**Figure 18:**
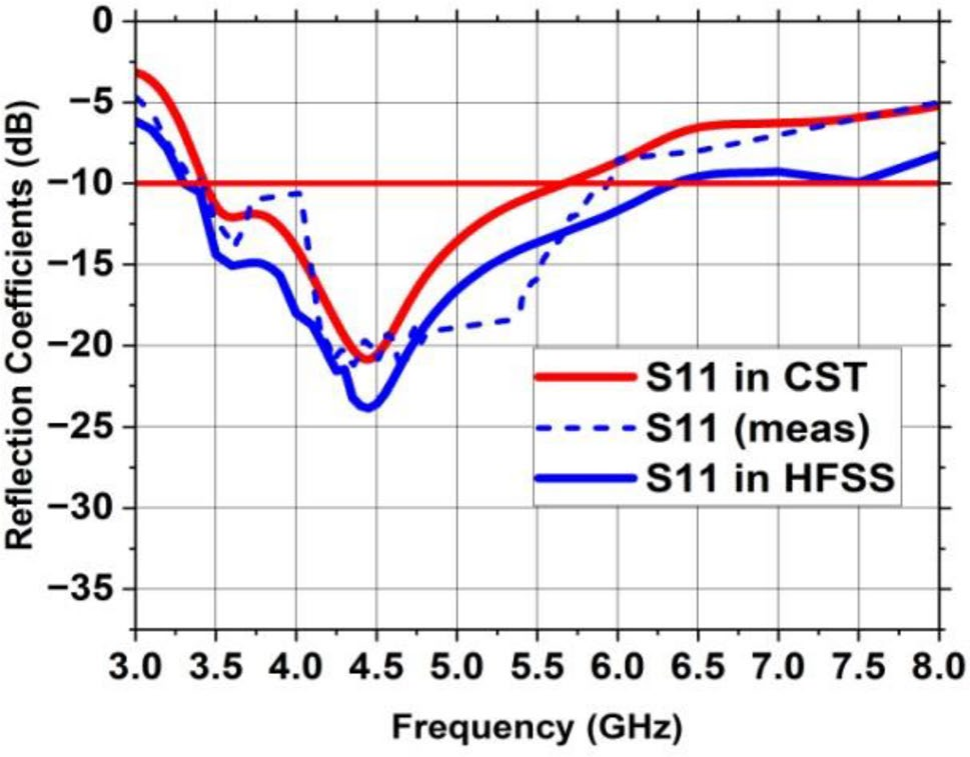
Reflection coefficient of the suggested MIMO antenna with a vertical strip.

**Figure 19:**
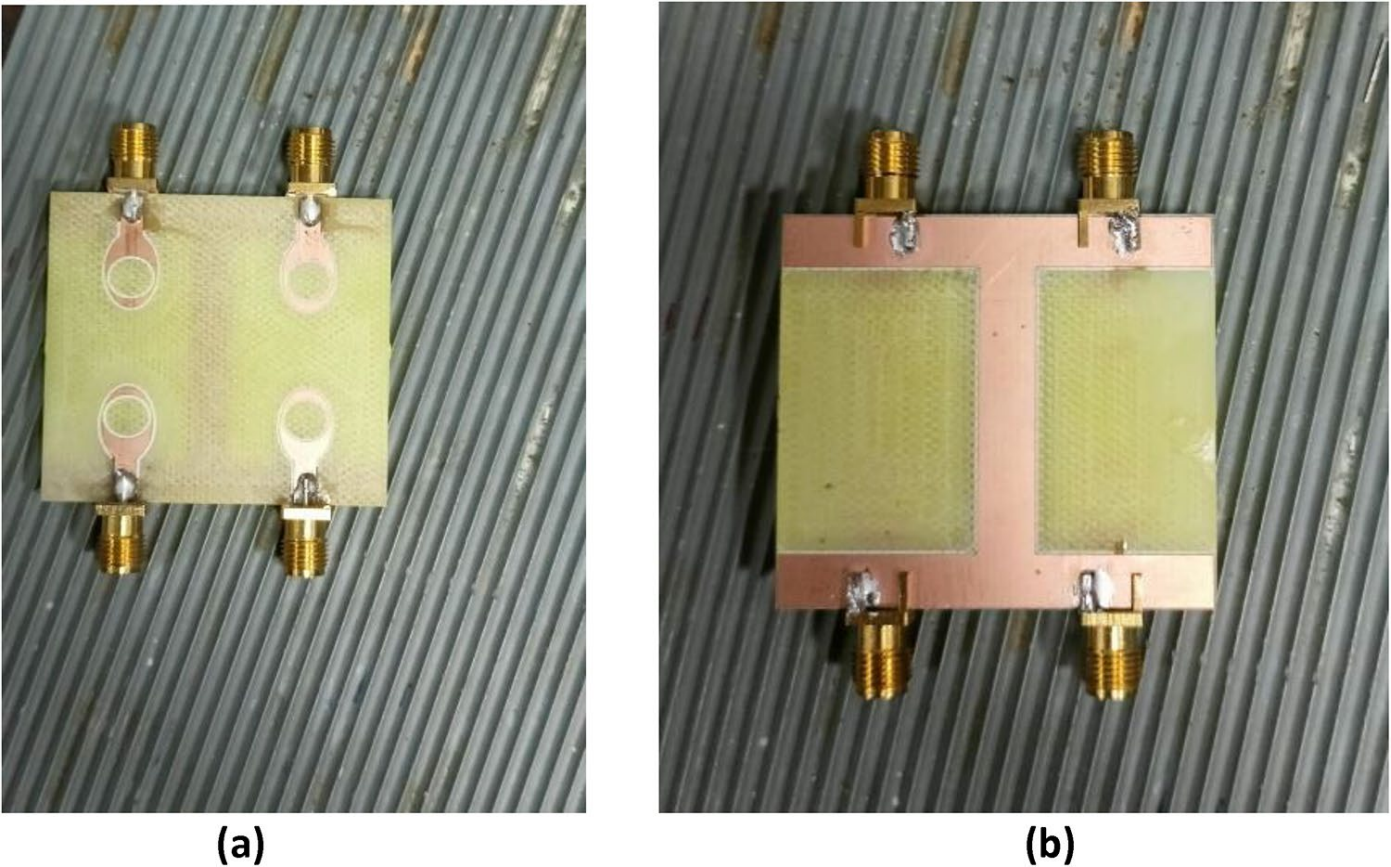
Fabricated MIMO antenna (**a**) Front side, (**b**) Back side.

MIMO antenna is displayed in Fig. 18. The MIMO antenna delivers a fractional bandwidth of 49.5% (3.4–5.6 GHz) with operating frequency of 4.44 GHz and return loss is − 20.8 dB. Near parity is discovered between the experimental and simulated reflection coefficients due to experimental tolerances. Furthermore, the suggested MIMO prototype’s observed reflection coefficient has wide impedance bandwidth of 2.2 GHz, encompassing the sub-6 GHz range of 5G band. The MIMO antenna was realized using the LPKF ProtoMat-S103 fabrication machine and is shown in Fig. 19. The S-parameter (S11) of the fabricated prototype was measured using a PNA Network Analyzer (Model: E5063A), which operates in the 1–40 GHz range. The radiation pattern, gain and efficiency of the design were measured using the SG-64 anechoic chambers provided by Microwave Vision Group.

**Figure 20:**
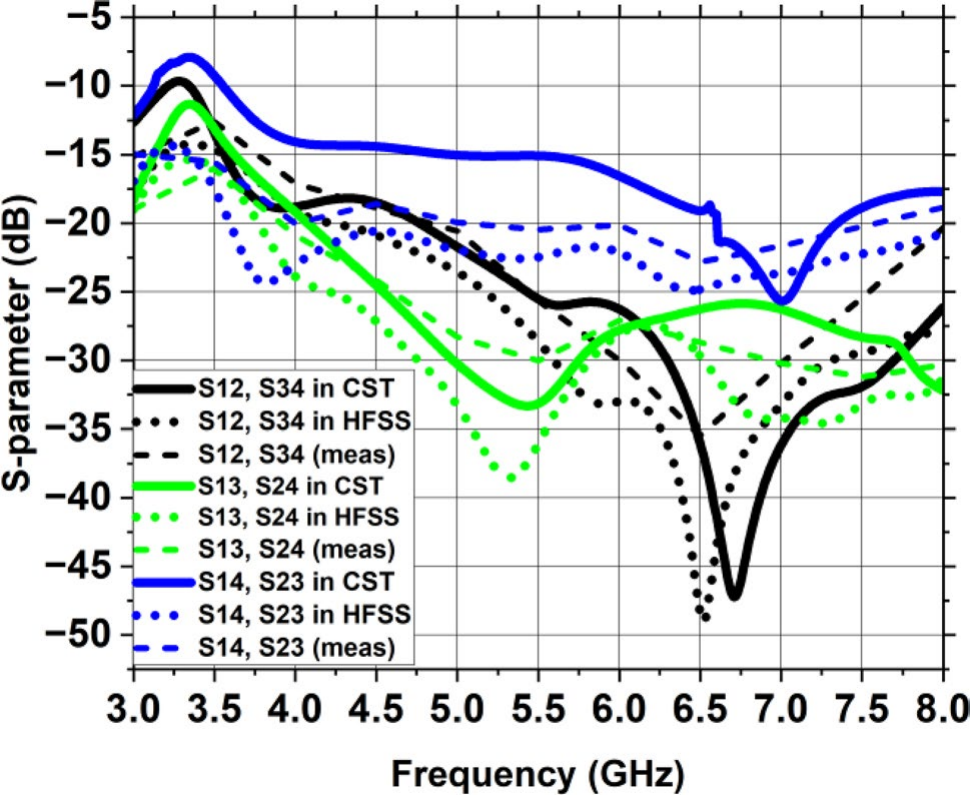
Transmission coefficient of the suggested MIMO antenna with a vertical strip.

Figure 20 displays the simulated and measured transmission coefficient curves of the MIMO architecture, demonstrating the mutual coupling between MIMO parts. This vertical strip, incorporated in the partial ground plane, serves as a decoupling channel and a reflector to the nearby fields. The length of the vertical strip should be approximately 1.2 times the length of the single antenna element to possess isolation level greater than 20 dB. Due to incorporation of vertical strip on the partial ground plane, the inter-element coupling is reduced to a greater extent. Similar trends can be seen in the graphs showing the isolation between close antenna parts S12, S34 and S14, S23 while identical high isolation can be observed between diagonal MIMO antennas S13 and S42. This image shows that experimental inter-element coupling between the MIMO constituents is quite close to the simulated coupling.

**Figure 21:**
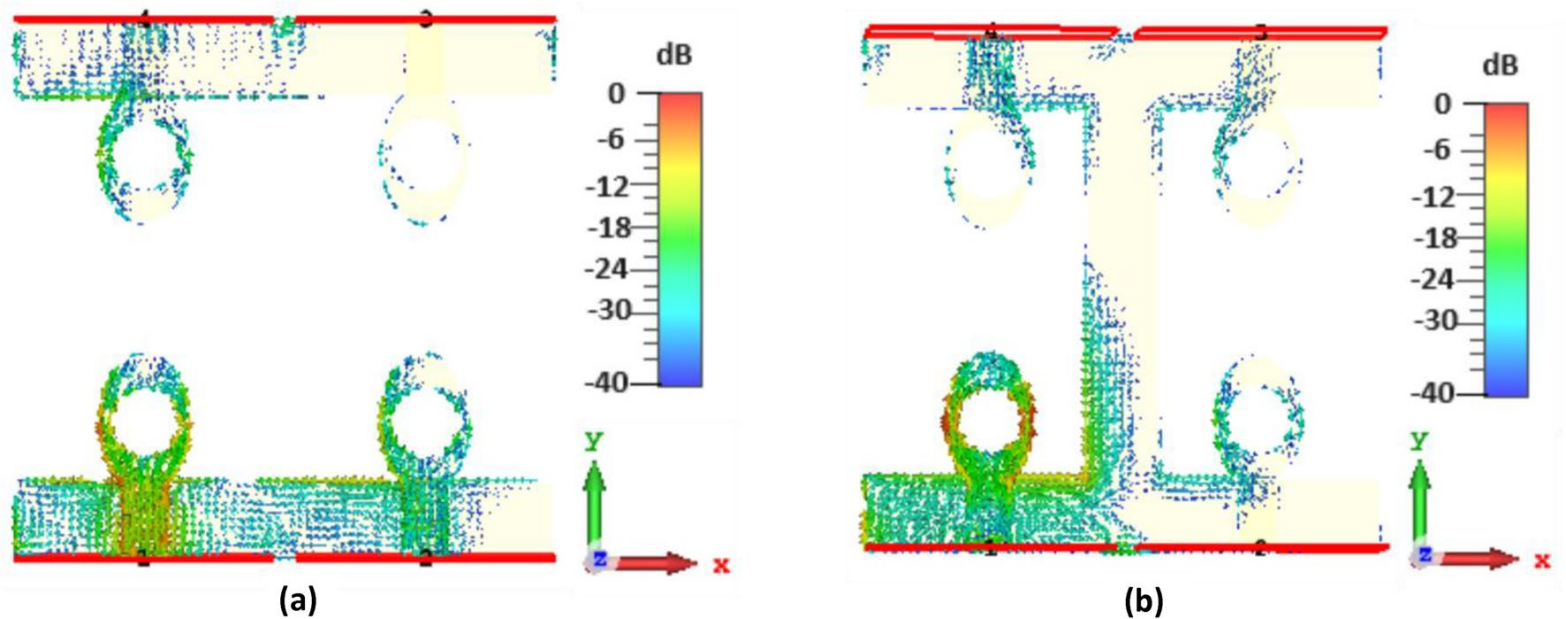
Surface current distribution of the MIMO antenna (**a**) excluding vertical strip, (**b**) including vertical strip.

The suggested MIMO antenna’s surface current distribution is depicted in Fig. 21 at operating frequency of 4.44 GHz where antenna 1 is activated and the remaining antennas are discontinued by a load of 50 Ω. When energy is supplied to antenna 1, a significant quantity of current emerges at the nearby antenna that causes inter-element coupling at 4.44 GHz excluding vertical strip as illustrated in Fig. 21a. In contrast, by using the vertical strip as shown in Fig. 21b, nearby antennas get more isolated from one another. The current’s distribution is greatest towards the elliptical patch’s margins. The optimization of the feedline and circular slot resulted in excellent impedance matching, which allowed the radiating patch to receive a significant percentage of the signal from the source. Consequently, the MIMO antenna’s radiation efficiency is enhanced. Furthermore, the created MIMO antenna has a radiation pattern that is omnidirectional and has a gain of 3.01 dBi overall.

**Figure 22:**
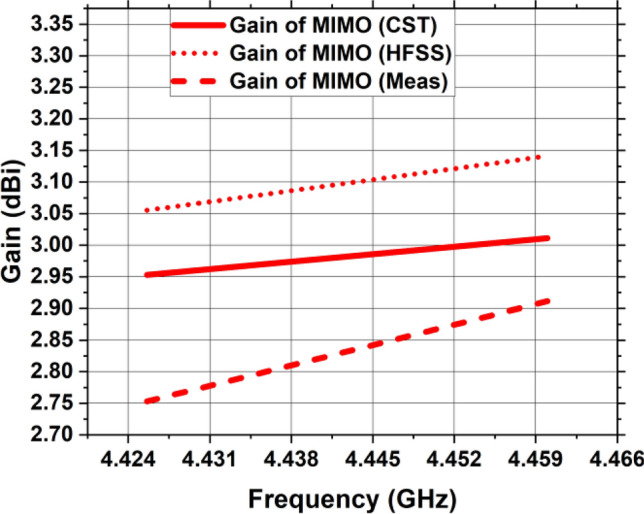
The suggested MIMO antenna’s gain.

**Figure 23:**
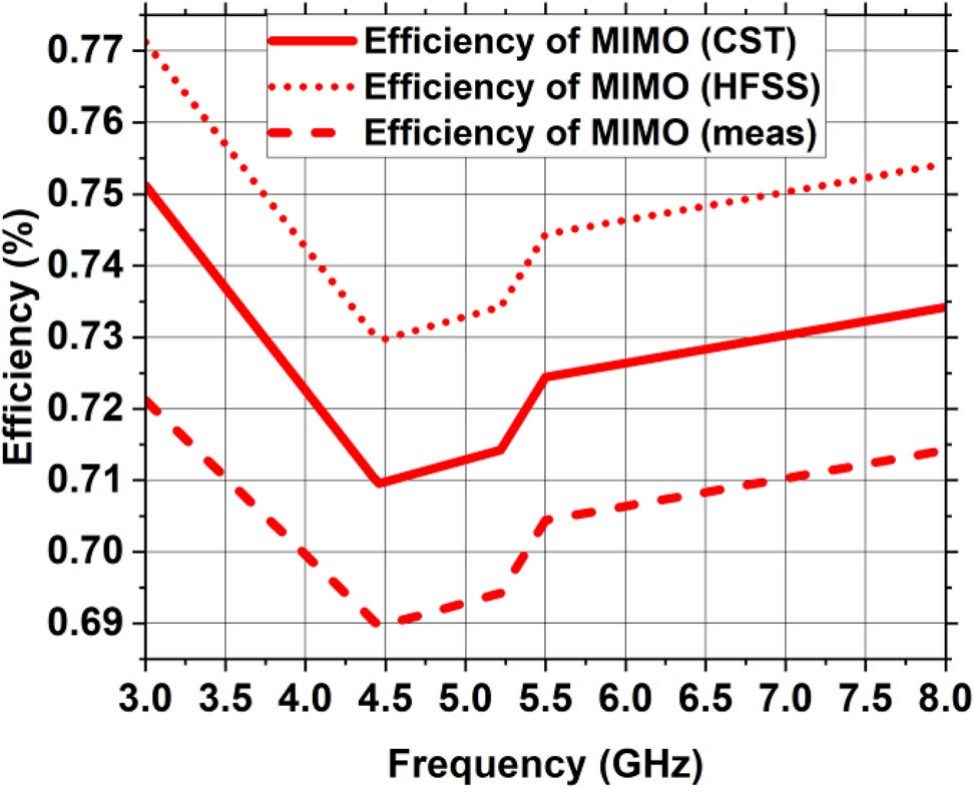
Efficiency of radiation of the suggested MIMO antenna.

**Figure 24:**
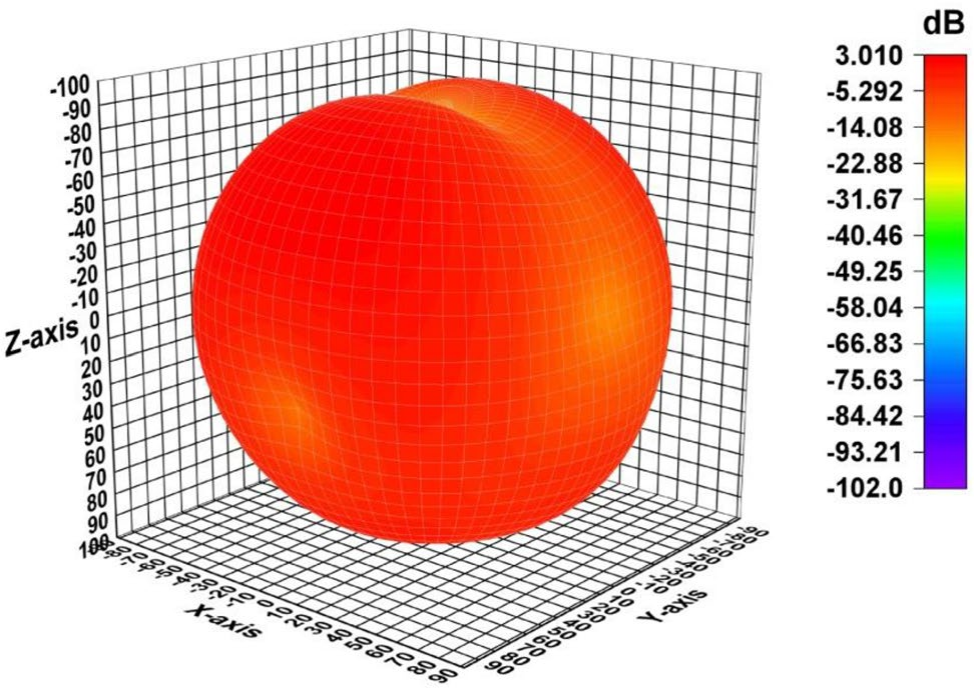
A 3D plot of the suggested MIMO antenna’s gain.

**Figure 25:**
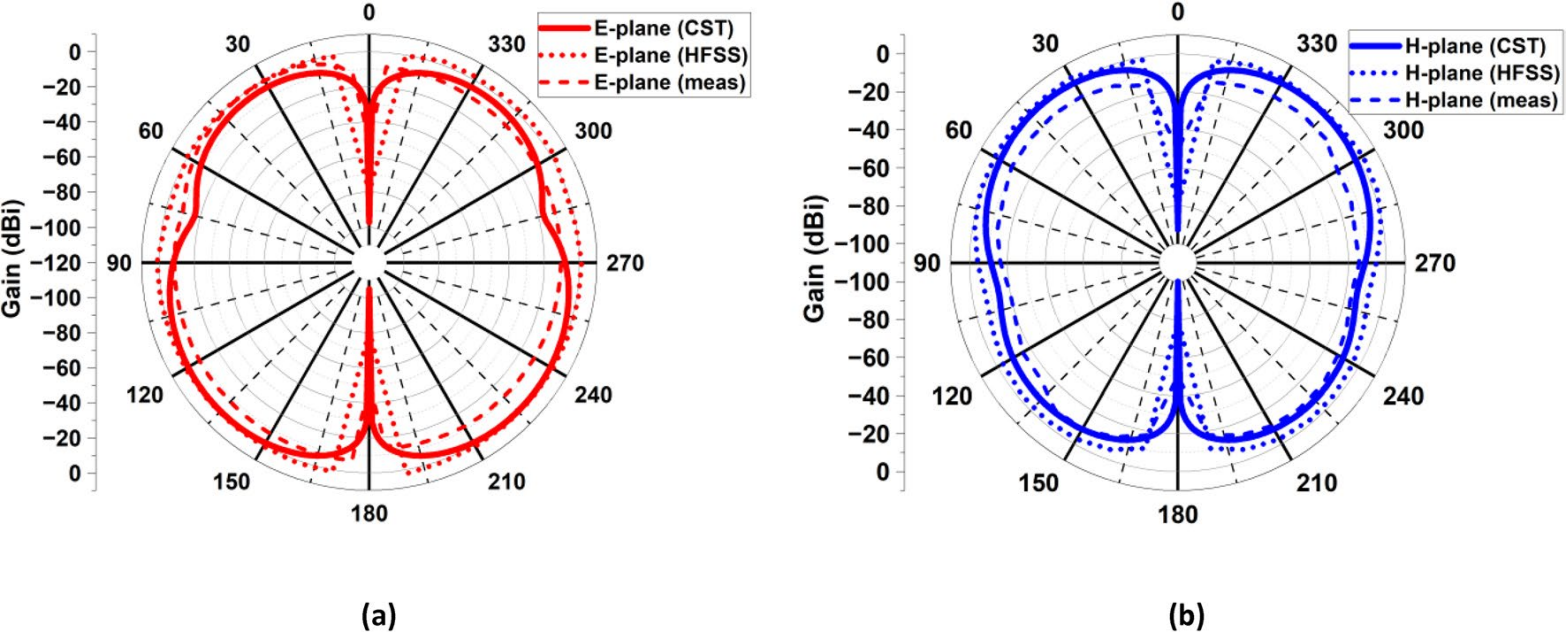
The 2D radiation configuration of the intended MIMO antenna at 4.44 GHz (**a**) in E-plane at $\varphi = {90}^{\circ}$, (**b**) in H-plane at $\varphi = {0}^{\circ}.$

Figure 22 depicts the constructed MIMO antenna’s achieved gain at 4.44 GHz operational frequency based on simulation and observation. The MIMO antenna has a computed actual gain of 3.01 dBi. The addition of vertical strip is responsible for the high gain along with outstanding seclusion. The fabricated MIMO antenna sample has nearly identical values of computed and observed gains. The variation in the actual gain is due to the errors in measurement and losses in cable. In both experimental and numerical settings, a high realized gain is demonstrated by the designed MIMO antenna configuration with four ports. Utilizing 4.44 GHz as the operational frequency, the MIMO system’s radiation efficiency, shown in Fig. 23, is 70%. At operational frequency, the actual gain and radiation efficiency closely match the results of the simulation, demonstrating that the intended MIMO antenna fits well with networks running on 5G, and the suggested MIMO.

architecture operates as anticipated. Figure 24 depicts the 3D gain pattern of 2 × 2 MIMO antenna at 4.44 GHz. The pattern is omnidirectional with gain of 3.01 dB. The predicted and observed distributions of radiation in the H- and E-planes for the suggested MIMO design at 4.44 GHz are depicted in Fig. 25. The MIMO design at its operating frequency has an omnidirectional radiation pattern. However, there is a slight irregularity in its pattern at $\varphi ={90}^{\circ}$ and $\varphi ={0}^{\circ}.$ This is because of the introduction of vertical strip in partial ground plane that makes the radiation to appear as a slight bump in its pattern. The radiation pattern was measured and collected from port 1, and the remaining ports were terminated due to 50Ω load. It is discovered that the CST generated radiation pattern and the one generated by experiment are almost identical.

It is imperative to emphasize that assessing a MIMO’s system performance should require knowledge of its port separation and correlating features. To demonstrate the robustness of the created MIMO antenna framework, performance in terms of diversity, including Envelope Correlation Coefficient (ECC), Diversity Gain (DG), and Mean Effective Gain (MEG), has been examined. These qualities are being demonstrated thoroughly with respect to the recommended antenna with MIMO technology.

In terms of their individual characteristics, the property of how well the component parts correspond with one another is determined by ECC when evaluating any MIMO system. It illustrates the great level of channel segregation in communication over wireless networks. To compute the ECC of the built-in MIMO arrangement, one can utilize the S-parameters and radiations in the far-field. ECC of the intended MIMO antenna architecture is assessed using Eqs. (19) and (20).

19 $${\rho }_{eij}=\frac{{\left|{S}_{ii}*{S}_{ij}+{S}_{ji}*{S}_{jj}\right|}^{2}}{\left(1-{\left|{S}_{ii}\right|}^{2}-{{S}_{jj}}^{2}\right)\left(1-{\left|{S}_{ji}\right|}^{2}-{{S}_{jj}}^{2}\right)}$$

20 $${\rho }_{eij}=\frac{\left|\int {\int }_{0}^{4\pi }\left[{\overrightarrow{R}}_{i}\left(\theta ,\varphi \right)*{\overrightarrow{R}}_{j}\left(\theta ,\varphi \right)\right]d\Omega \right|}{\int {{\int }_{0}^{4\pi }\left|{\overrightarrow{R}}_{i}\left(\theta ,\varphi \right)\right|}^{2}d\Omega \int {{\int }_{0}^{4\pi }\left|{\overrightarrow{R}}_{j}\left(\theta ,\varphi \right)\right|}^{2}d\Omega }$$

**Figure 26:**
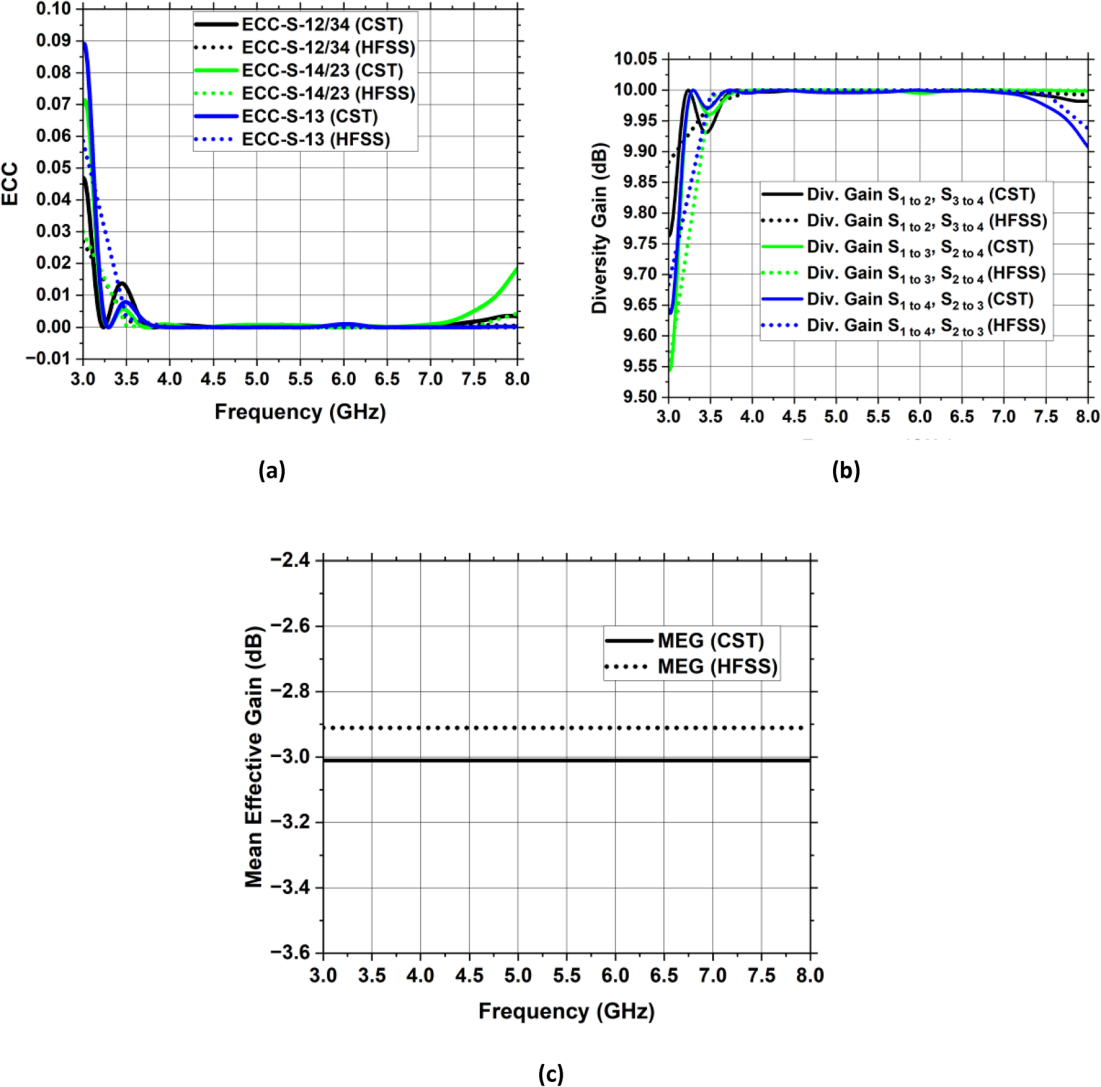
Diversity-related MIMO design characteristics (**a**) ECC, (**b**) DG, (**c**) MEG.

${S}_{ii}$ denotes the reflection coefficient and ${S}_{jj}$ denotes the transmission coefficient. ${\overrightarrow{R}}_{i}\left(\theta ,\varphi \right)$ and ${\overrightarrow{R}}_{j}\left(\theta ,\varphi \right)$ depicts three- dimensional pattern of radiation for ${i} {\text{th}}$ and ${j} {\text{th}}$ antenna while solid angle is denoted by Ω. Figure 26a depicts the ECC map of the suggested antenna having values less than 0.01 which is much less than the allowable value of 0.5 for IoT systems. As a result of the lower ECC value, the proposed four-port MIMO system delivers a superior diversity pattern.

The Diversity Gain (DG) is a further statistic of MIMO system efficiency which illustrates the effect of diversity scheme on the emitted energy, as described in^34^, the designed MIMO antenna framework’s DG is defined by the formula below.

21 $$DG=10\sqrt{{1-\left|{\rho }_{eij}\right|}^{2}}$$

Figure 26b displays the proposed MIMO architecture’s DG curve. DG has a value of 9.99 dB, which is near to the 10 dB threshold.

MEG gauges the proportion of the mean power acquired by an antenna to its mean incident power while accounting for environmental effects. It is possible to compute MEG using Eqs. (22) and (23).

22 $${MEG}_{i}=0.5\left(1-{\sigma }_{j=1}^{k}\left|{S}_{ij}\right|\right)$$

23 $${MEG}_{ij}={MEG}_{i}-{MEG}_{j}$$

where $k$ is the MIMO design’s entirety of antenna components.

Figure 26c depicts the MEG of the envisioned MIMO antenna framework which is – 3 dB for all the ports and it is within the range as defined by^35^.

Comparison with the state-of-the-art designs is given in Table 3.

**Table 3:** Comparative analysis with the state-of-the-art similar work.

Ref.	Year	Antenna dimensions (mm^3^)	No. of resonators	Freq (GHz)	S11 (dB)	BW (GHz)	Gain (dBi)	Max. isolation (dB)	ECC	DG (dB)
^36^	2023	90 × 90 × 1.6	4	5.9	− 40	0.5	–	30	< 0.02	9.98
^37^	2023	38 × 32 × 1.6	4	4	− 20	2	3.14	20	< 0.03	–
^38^	2023	64 × 64 × 1.6	4	5.8	− 25	0.45	–	25	< 0.04	9.8
^39^	2023	60 × 60 × 1.6	4	2.4	− 22.45	0.065	2.63	23	1.12 × 10^–7^	9.99
^40^	2023	180 × 180 × 1.575	4	3.24	− 30	0.04	4.14	25	< 0.2	–
^41^	2024	43 × 40 × 1.6	4	4.97	− 15	0.32	1.2	30	< 0.4	9.8
^42^	2024	80 × 80 × 1.6	4	5.9	− 30	0.17	6.3	35	0.028	9.98
Proposed	2024	44 × 44 × 1.6	4	4.44	− 20.85	2.2	3.01	38.53	< 0.01	9.99

## Conclusion

The 5G sub-6 GHz uses are supported by a quad-element MIMO antenna design that has a significant gain coupled with excellent isolation. A microstrip feedline energizing a patch with an elliptical form with a circular aperture carved out of it. The suggested antenna with MIMO technology is constructed using the FR-4 substrate to attain optimal efficiency in 5G wireless networks. The MIMO design stands out for its extensive coverage and strong gain, as well as its good efficiency and strong segregation amongst MIMO parts. The individual antenna component is small with impedance bandwidth of 2 GHz, operating frequency is 4.33 GHz, gain is 1.81 dBi and 75% radiation efficiency as the findings illustrate. Each suggested individual antenna is positioned in an upright manner to form the recommended MIMO design with four antenna elements. Based on the outcomes of the investigation and modeling, the built-in MIMO model runs over a wide frequency band, addressing the 5G sub-6 GHz band, having the spectrum of 3.4 to 5.6 GHz. Moreover, the MIMO antenna that is currently under proposal has maximum gain of 3.01 dBi and provides a substantial amount of separation between MIMO parts (> 20 dB) due to incorporation of vertical strip in partial ground plane and optimal gap between the antenna modules. Furthermore, the recommended MIMO antenna has a high average overall efficiency of 70%. The antenna displayed exceptional MIMO diversity properties with a high DC (9.98 dB), minimal ECC (less than 0.01) and a radiation behavior of omnidirectional.

## Data Availability

The datasets generated and/or analyzed during the current study are available from the corresponding author upon reasonable request.
